# Supplementary material for: Progressive unanchoring of Antarctic ice shelves since 1973
Source: Nature. 2024 Feb 21;626(8000):785–91. doi: 10.1038/s41586-024-07049-0 (PMC10881387; doi:10.1038/s41586-024-07049-0)
Supplement: Supplementary file 1 — Supplementary Fig. 1 and Supplementary Table 1 [file 41586_2024_7049_MOESM1_ESM.pdf]

nature portfolio

https://doi.org/10.1038/s41586-024-07049-0

Supplementary information

Progressive unanchoring of Antarctic ice shelves since 1973

In the format provided by the authors and unedited

Supplementary Information

Examples of pinning point change. The multi panel figure shows Landsat imagery from 1973, 1989, 2000 and 2022 over a selection of 108 pinning points. Mapping of pinning point change is overlain. Black crosses represent no change, red crosses represent a reduction in pinning point size and blue crosses represent an increase in size. The location of each pinning point and name, where available, is included in each heading. The ID number is the identification number provided in Matsuoka et al. (2015) pinning point database, pinning points that were not included in the Matsuoka et al. database are labelled as ‘ID N/A’.

Landsat scenes used to create mosaics. A list of all Landsat scene ID’s used in 1973 and 1989 mosaics of Antarctic ice shelves.

Supplementary Data: Animated images of pinning point change. A series of animated imagery flicking between 1973, 1989, 2000 and 2022 over key ice shelves and pinning points.

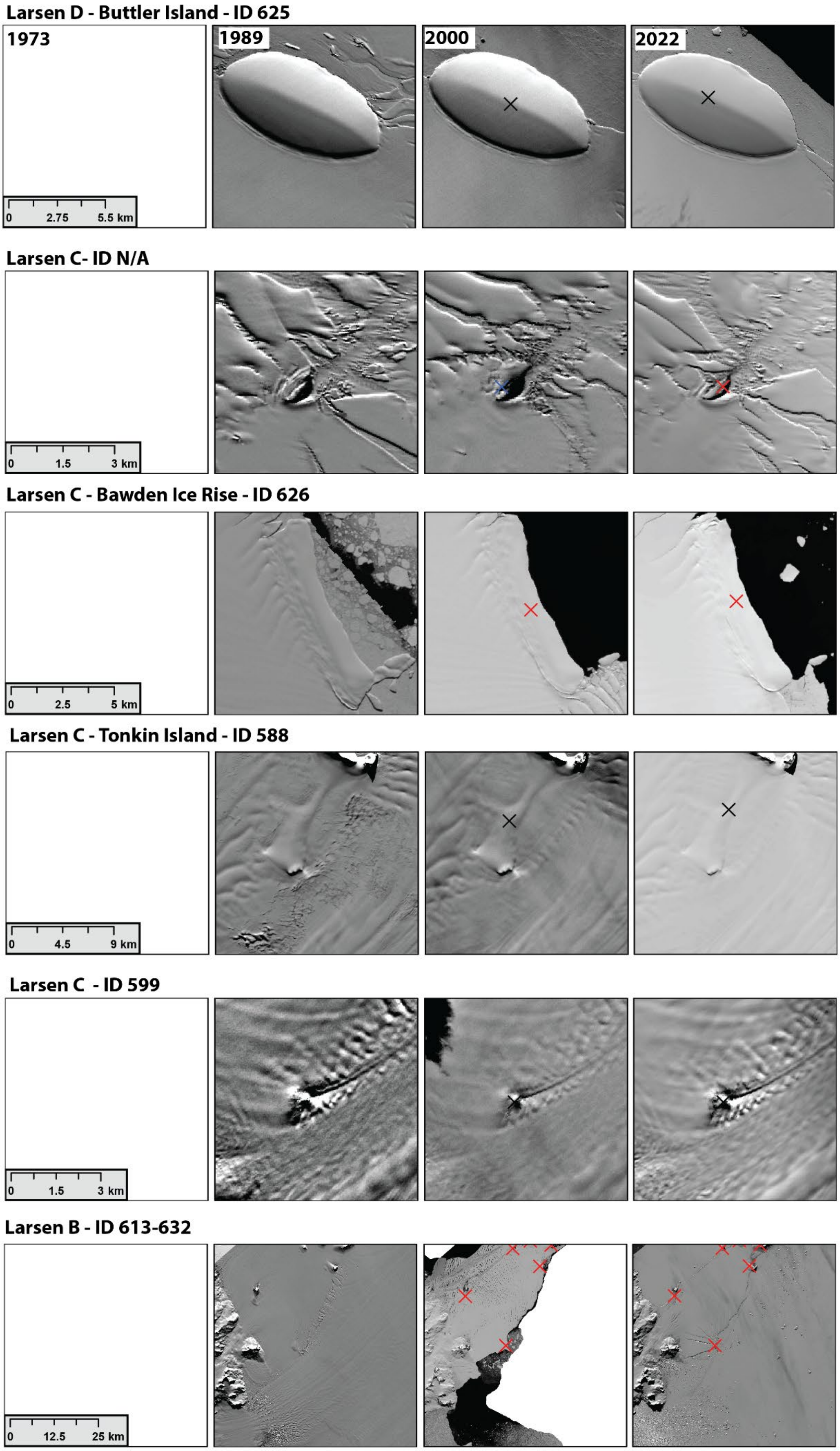
Larsen D - Buttler Island - ID 625
1973
1989
2000
2022
0 2.75 5.5 km
Larsen C- ID N/A
0 1.5 3 km
Larsen C - Bawden Ice Rise - ID 626
0 2.5 5 km
Larsen C - Tonkin Island - ID 588
0 4.5 9 km
Larsen C - ID 599
0 1.5 3 km
Larsen B - ID 613-632
0 12.5 25 km

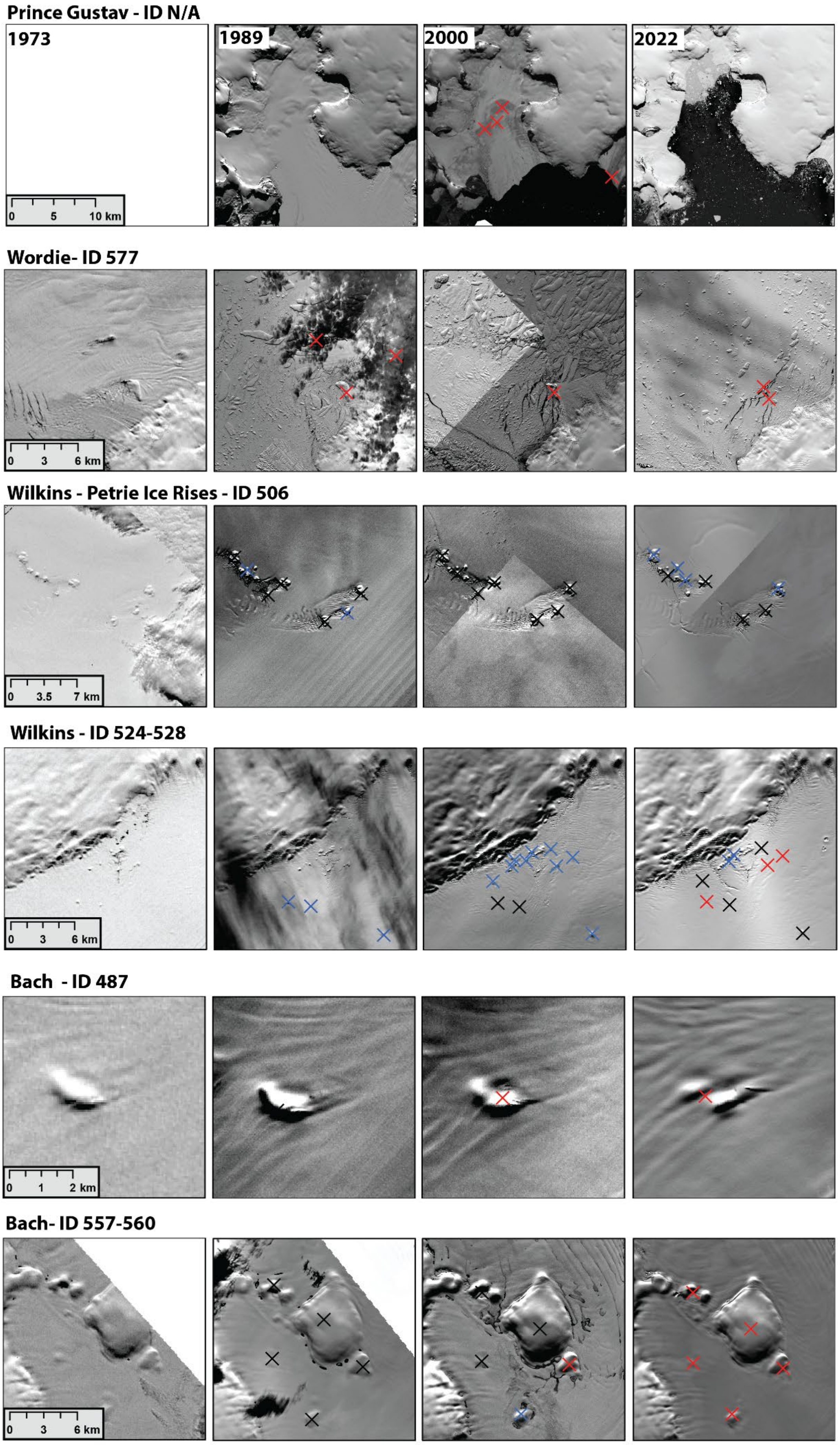

Prince Gustav - ID N/A
1973
1989
2000
2022
0 5 10 km
Wordie- ID 577
0 3 6 km
Wilkins - Petrie Ice Rises - ID 506
0 3.5 7 km
Wilkins - ID 524-528
0 3 6 km
Bach - ID 487
0 1 2 km
Bach- ID 557-560
0 3 6 km

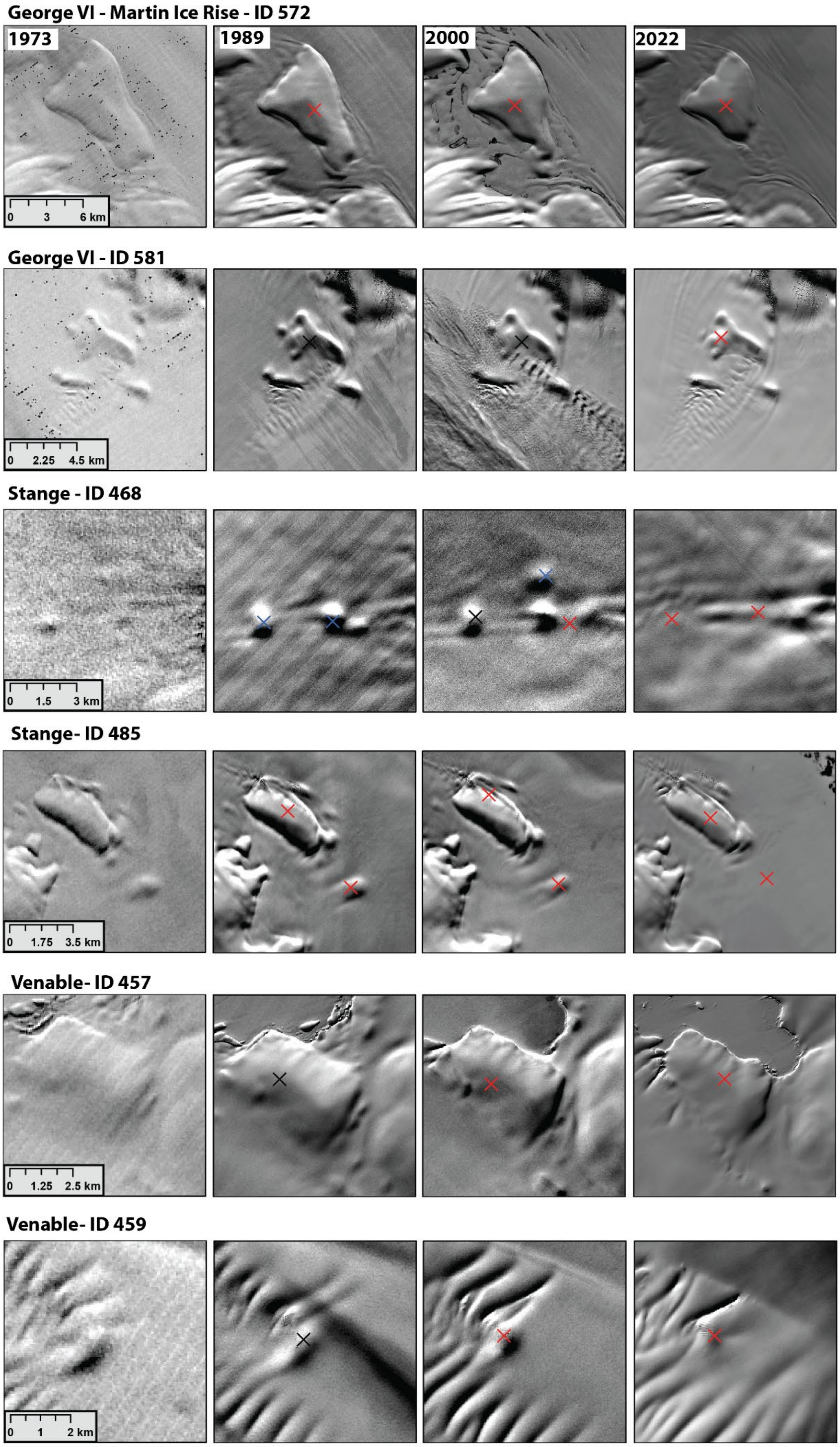

George VI - Martin Ice Rise - ID 572
1973
1989
2000
2022
0 3 6 km
George VI - ID 581
0 2.25 4.5 km
Stange - ID 468
0 1.5 3 km
Stange- ID 485
0 1.75 3.5 km
Venable- ID 457
0 1.25 2.5 km
Venable- ID 459
0 1 2 km

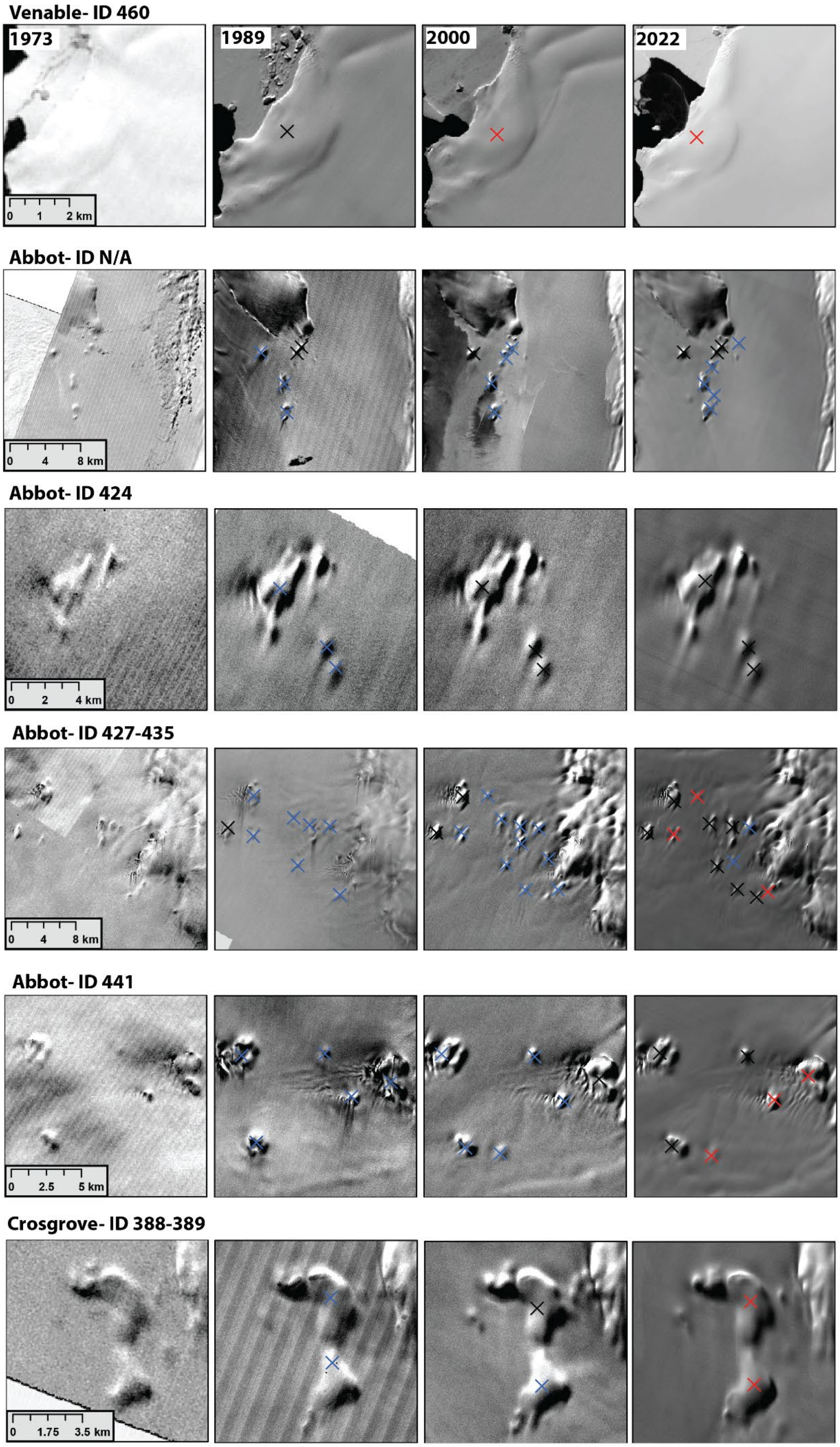
Venable- ID 460
1973
1989
2000
2022
0 1 2 km
Abbot- ID N/A
0 4 8 km
Abbot- ID 424
0 2 4 km
Abbot- ID 427-435
0 4 8 km
Abbot- ID 441
0 2.5 5 km
Crosgrove- ID 388-389
0 1.75 3.5 km

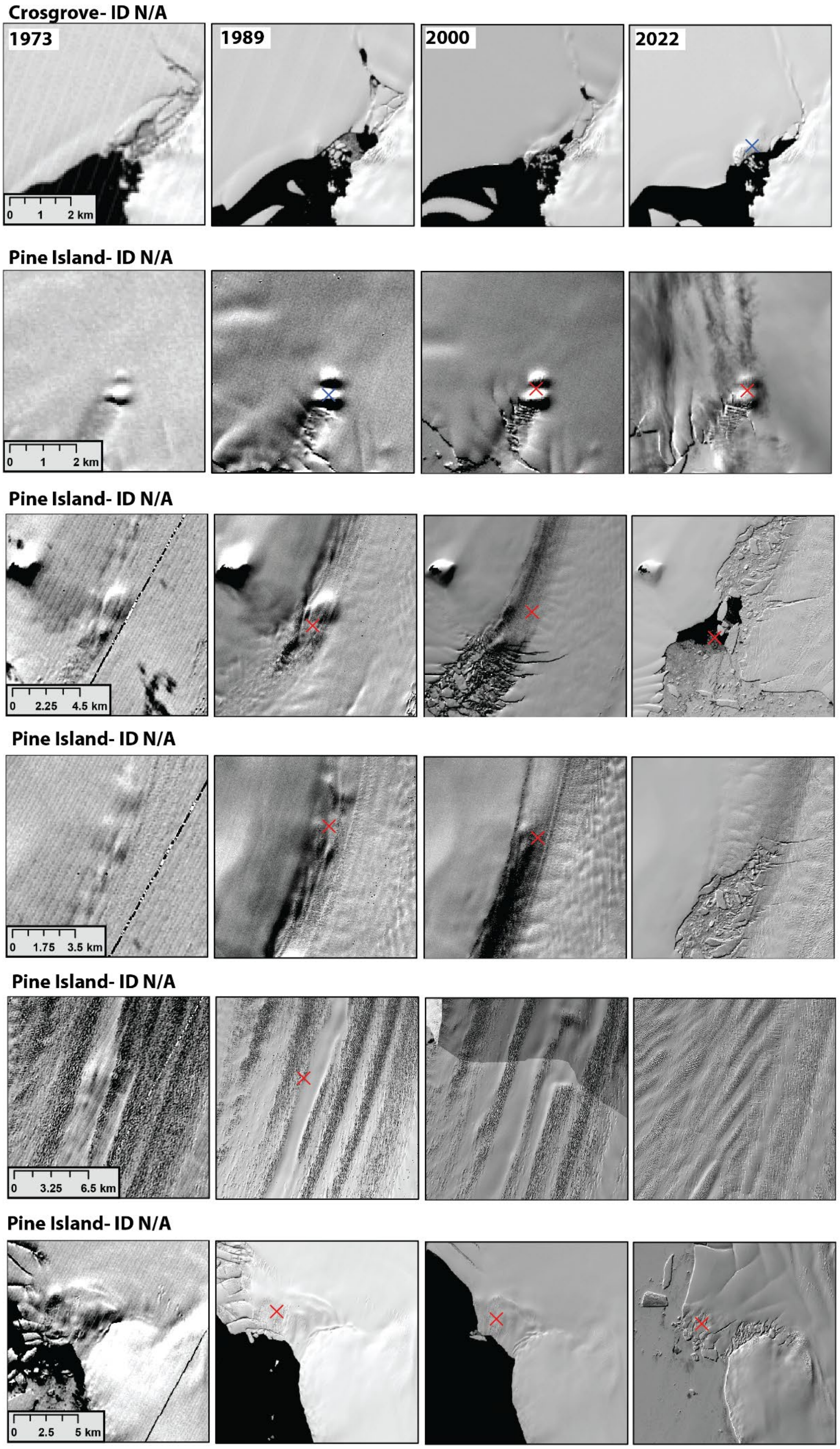
Crosgrove- ID N/A
1973
1989
2000
2022
0 1 2 km
Pine Island- ID N/A
0 1 2 km
Pine Island- ID N/A
0 2.25 4.5 km
Pine Island- ID N/A
0 1.75 3.5 km
Pine Island- ID N/A
0 3.25 6.5 km
Pine Island- ID N/A
0 2.5 5 km

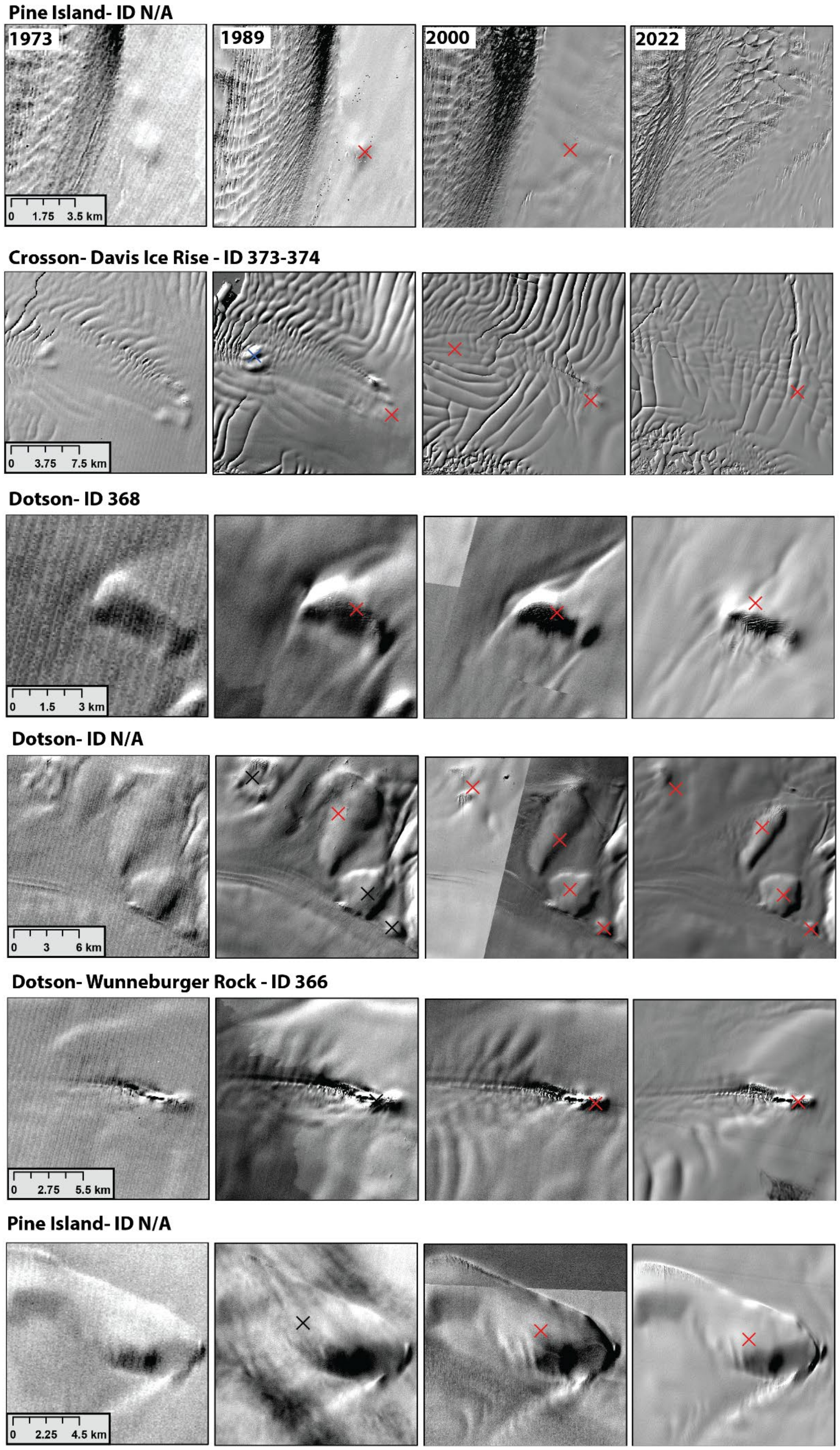

Pine Island- ID N/A
1973
1989
2000
2022
0 1.75 3.5 km
Crosson- Davis Ice Rise - ID 373-374
0 3.75 7.5 km
Dotson- ID 368
0 1.5 3 km
Dotson- ID N/A
0 3 6 km
Dotson- Wunneburger Rock - ID 366
0 2.75 5.5 km
Pine Island- ID N/A
0 2.25 4.5 km

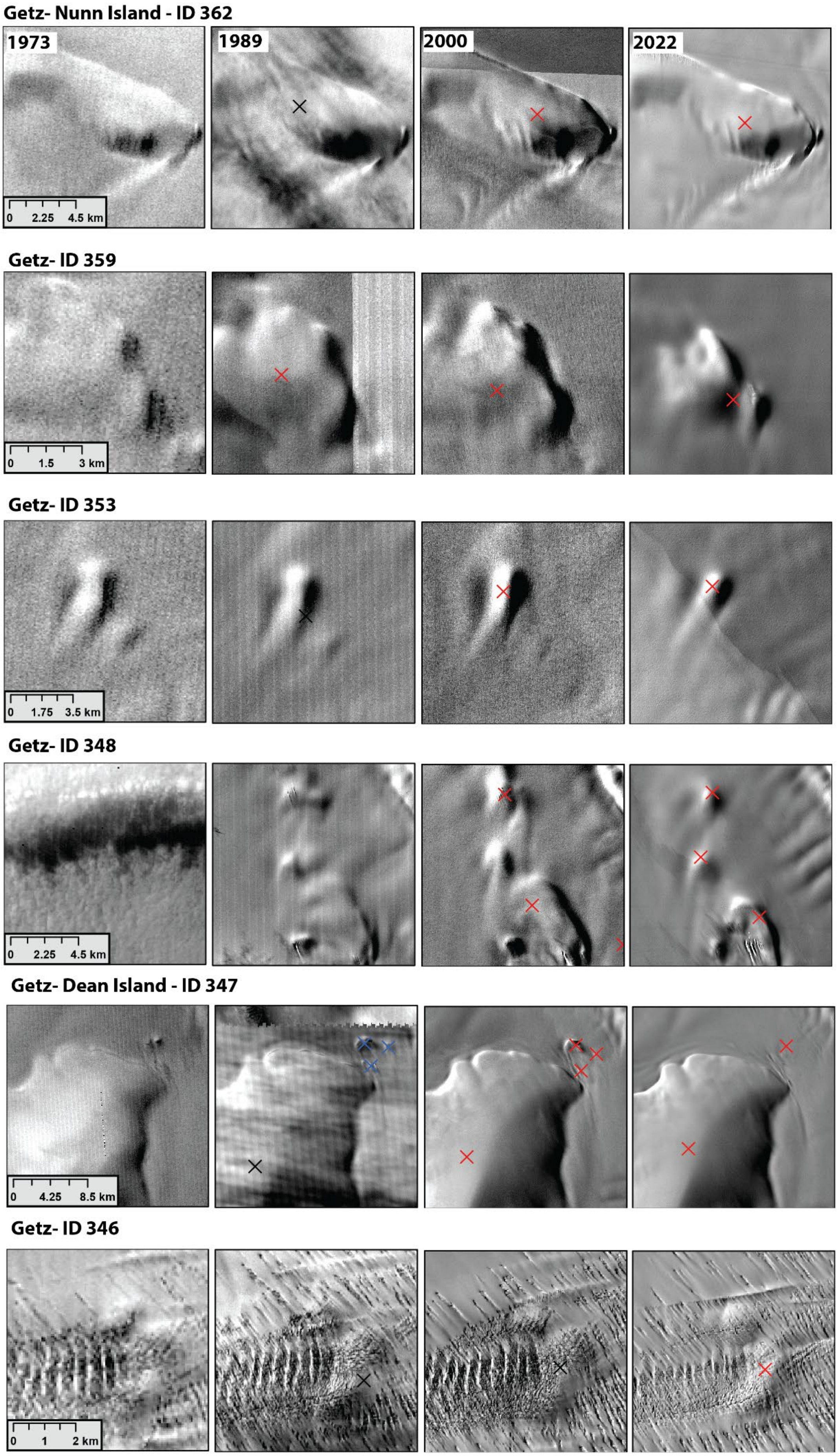
Getz- Nunn Island - ID 362
1973
1989
2000
2022
0 2.25 4.5 km
Getz- ID 359
0 1.5 3 km
Getz- ID 353
0 1.75 3.5 km
Getz- ID 348
0 2.25 4.5 km
Getz- Dean Island - ID 347
0 4.25 8.5 km
Getz- ID 346
0 1 2 km

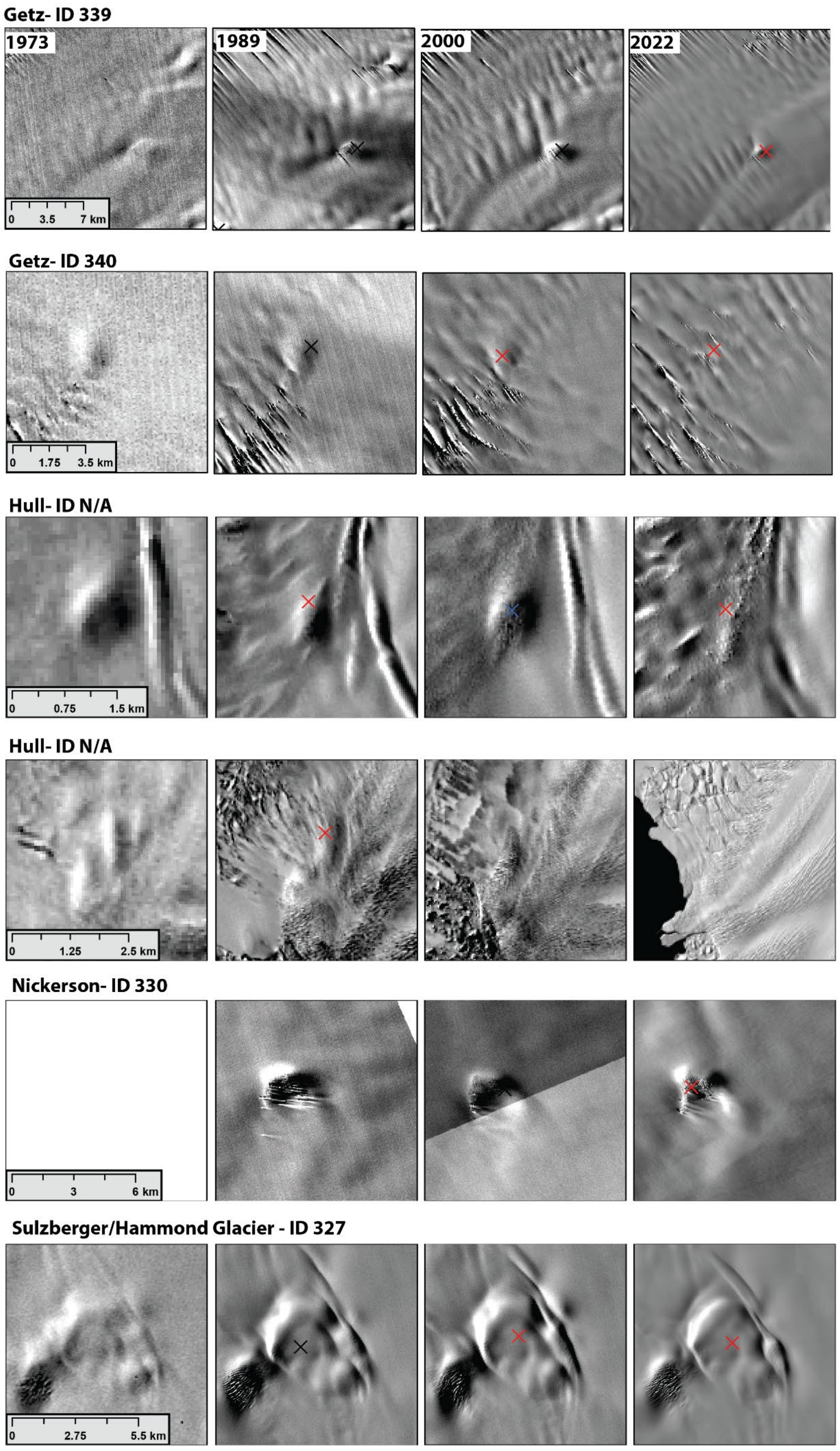
Getz- ID 339
1973
1989
2000
2022
0 3.5 7 km
Getz- ID 340
0 1.75 3.5 km
Hull- ID N/A
0 0.75 1.5 km
Hull- ID N/A
0 1.25 2.5 km
Nickerson- ID 330
0 3 6 km
Sulzberger/Hammond Glacier - ID 327
0 2.75 5.5 km

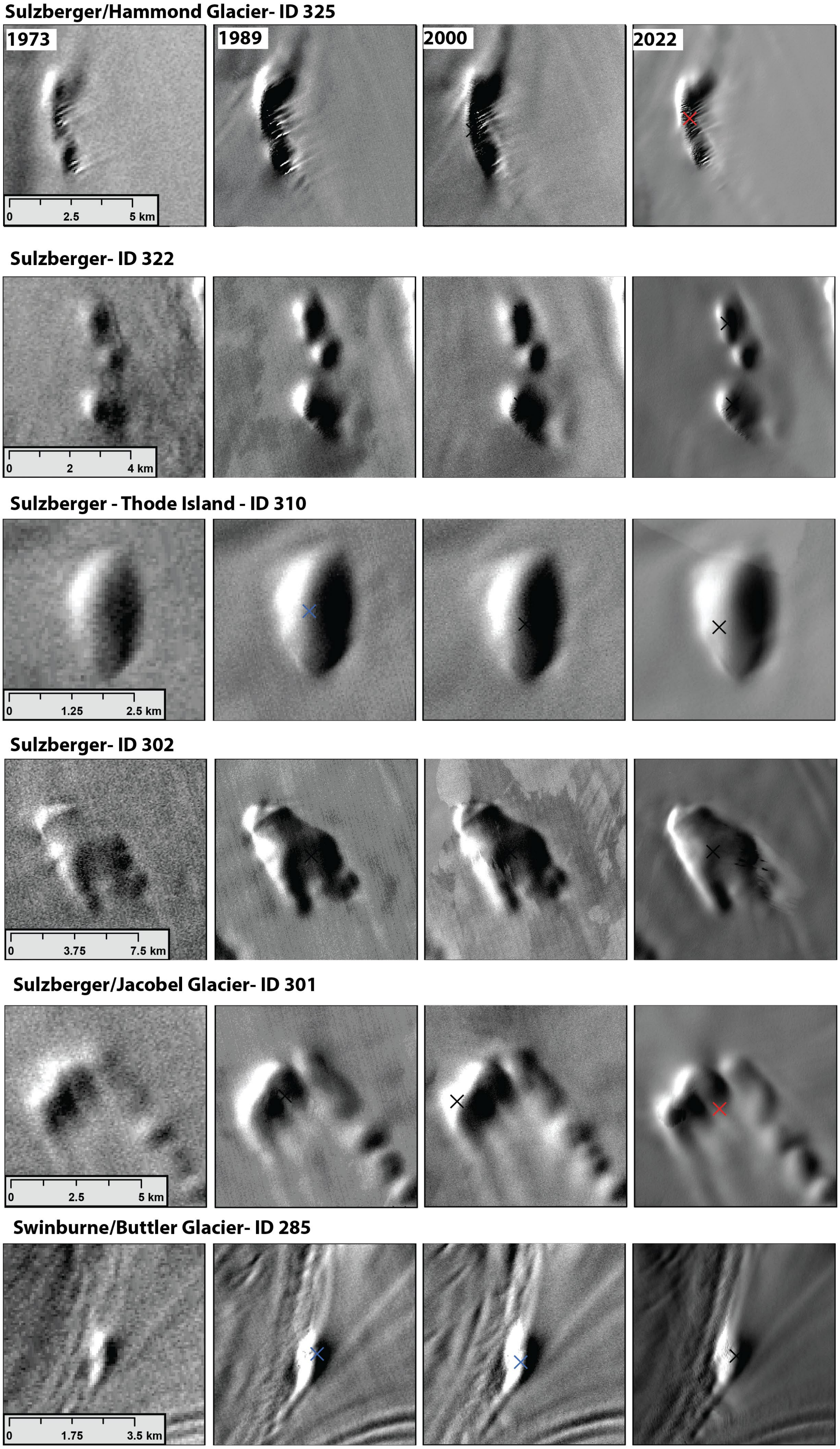
Sulzberger/Hammond Glacier- ID 325
1973
1989
2000
2022
0 2.5 5 km
Sulzberger- ID 322
0 2 4 km
Sulzberger - Thode Island - ID 310
0 1.25 2.5 km
Sulzberger- ID 302
0 3.75 7.5 km
Sulzberger/Jacobel Glacier- ID 301
0 2.5 5 km
Swinburne/Buttler Glacier- ID 285
0 1.75 3.5 km

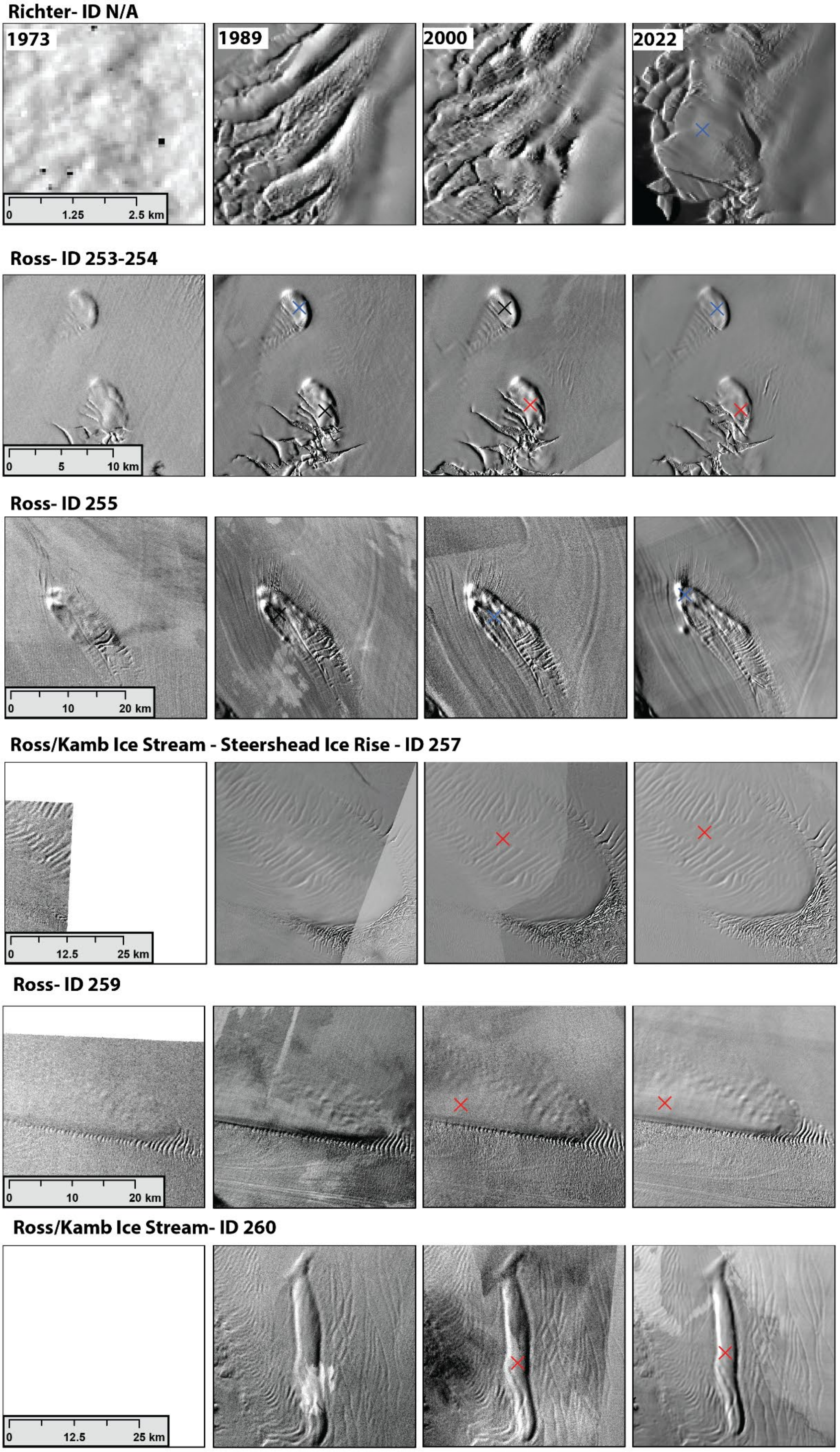
Richter- ID N/A
1973
1989
2000
2022
0 1.25 2.5 km
Ross- ID 253-254
0 5 10 km
Ross- ID 255
0 10 20 km
Ross/Kamb Ice Stream - Steershead Ice Rise - ID 257
0 12.5 25 km
Ross- ID 259
0 10 20 km
Ross/Kamb Ice Stream- ID 260
0 12.5 25 km

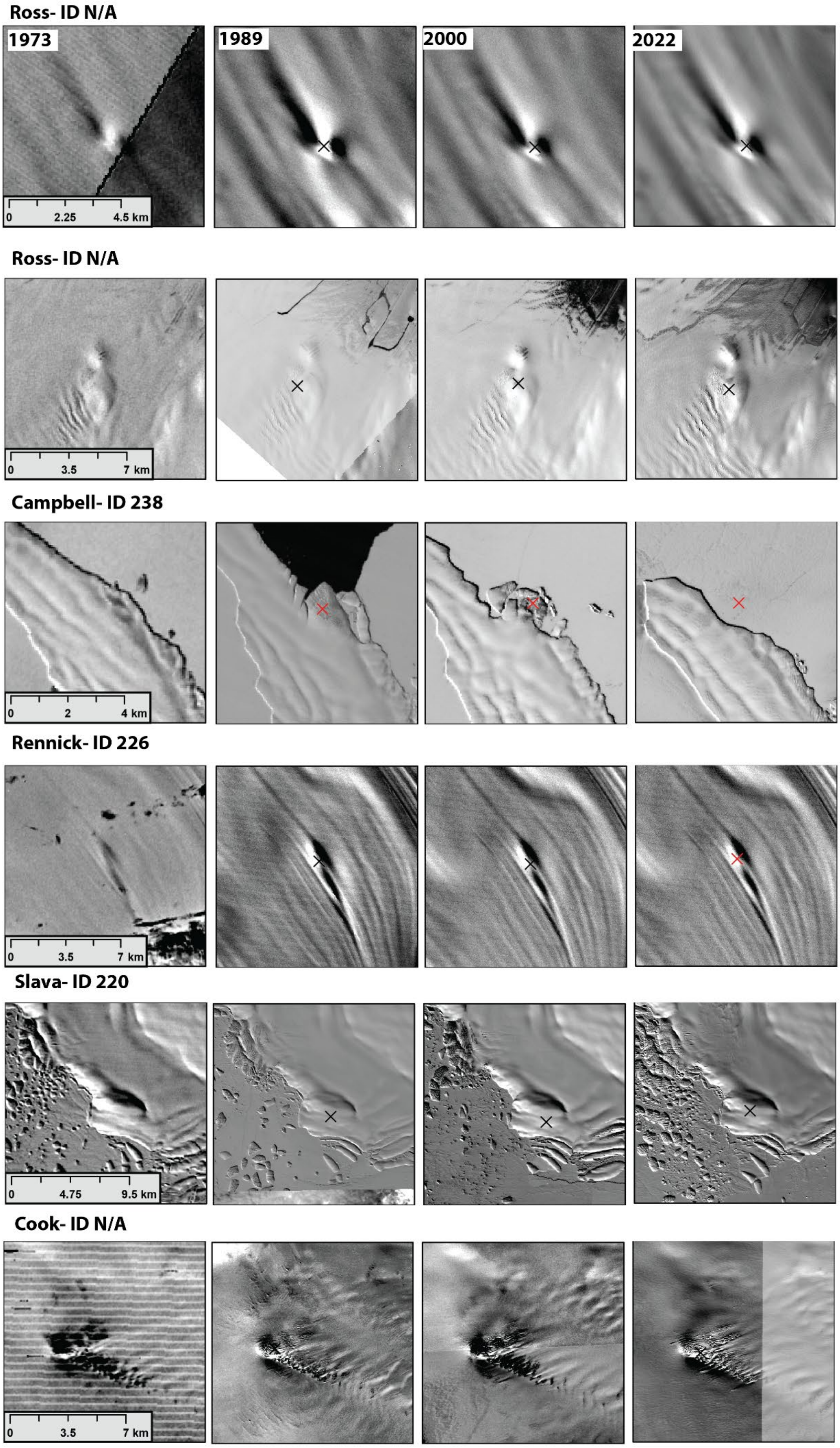
Ross- ID N/A
1973
1989
2000
2022
0 2.25 4.5 km
Ross- ID N/A
0 3.5 7 km
Campbell- ID 238
0 2 4 km
Rennick- ID 226
0 3.5 7 km
Slava- ID 220
0 4.75 9.5 km
Cook- ID N/A
0 3.5 7 km

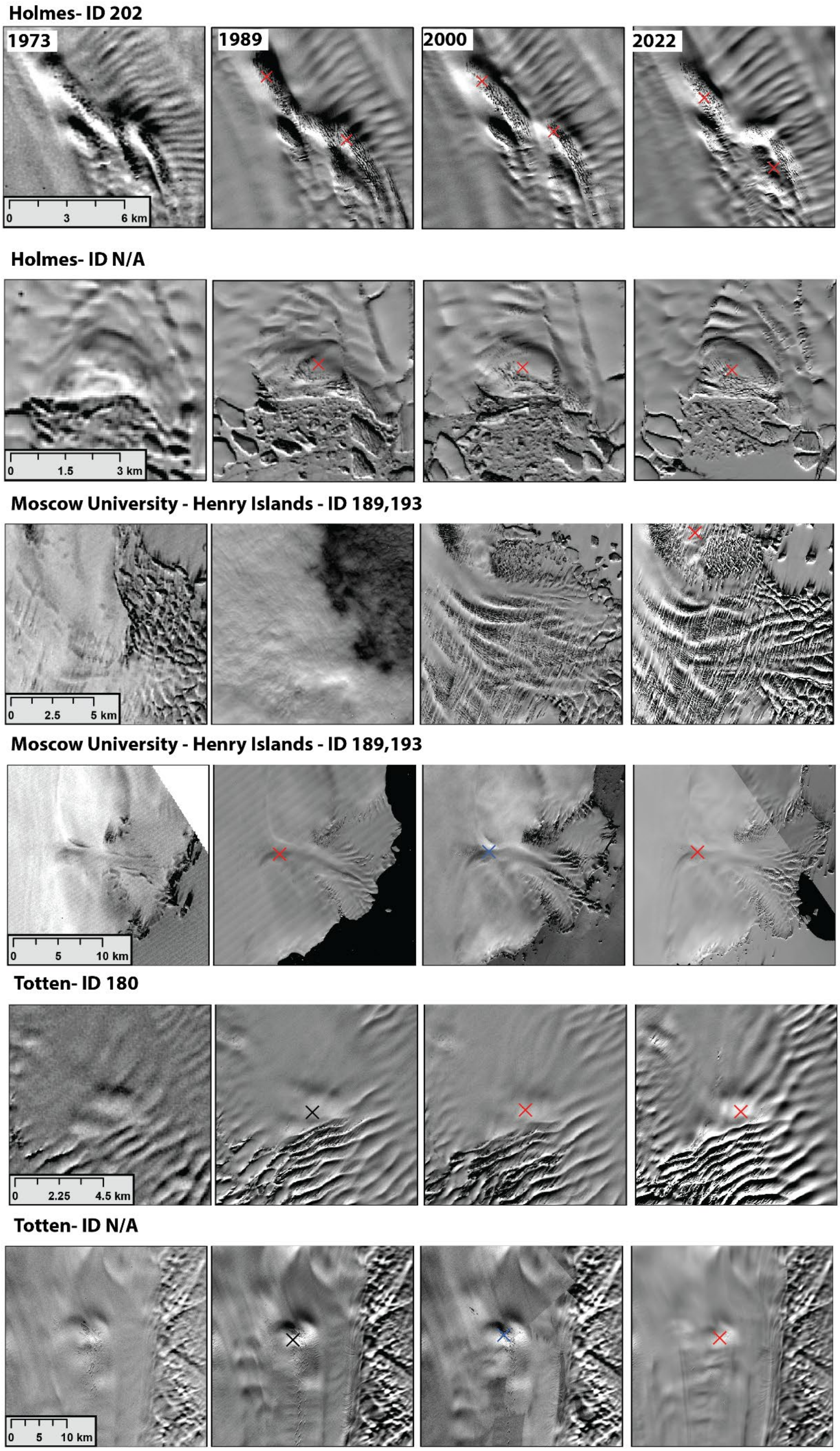
Holmes- ID 202
1973
1989
2000
2022
0 3 6 km
Holmes- ID N/A
0 1.5 3 km
Moscow University - Henry Islands - ID 189,193
0 2.5 5 km
Moscow University - Henry Islands - ID 189,193
0 5 10 km
Totten- ID 180
0 2.25 4.5 km
Totten- ID N/A
0 5 10 km

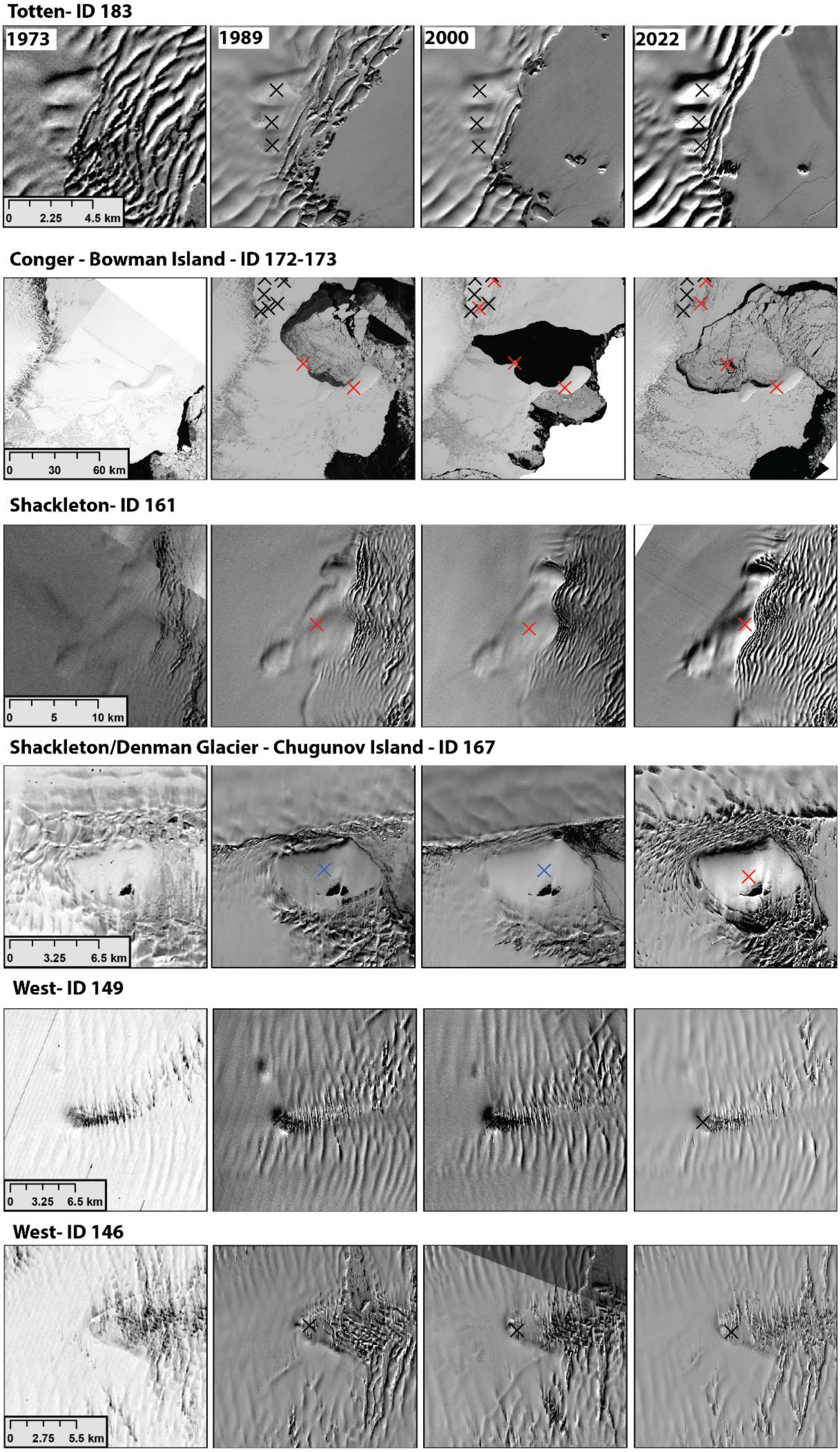
Totten- ID 183
1973
1989
2000
2022
0 2.25 4.5 km
Conger - Bowman Island - ID 172-173
0 30 60 km
Shackleton- ID 161
0 5 10 km
Shackleton/Denman Glacier - Chugunov Island - ID 167
0 3.25 6.5 km
West- ID 149
0 3.25 6.5 km
West- ID 146
0 2.75 5.5 km

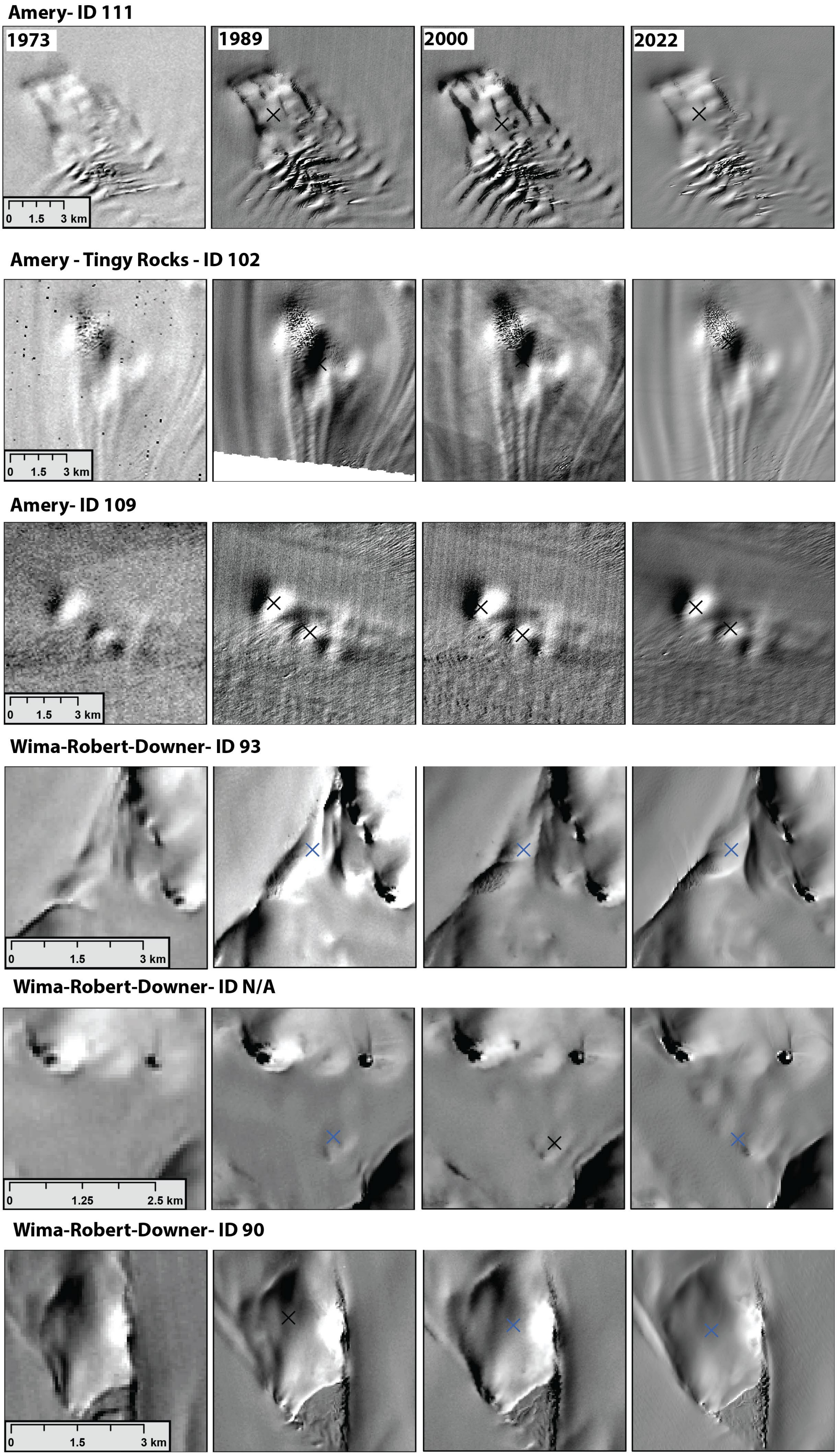
Amery- ID 111
1973
1989
2000
2022
0 1.5 3 km
Amery - Tingy Rocks - ID 102
0 1.5 3 km
Amery- ID 109
0 1.5 3 km
Wima-Robert-Downer- ID 93
0 1.5 3 km
Wima-Robert-Downer- ID N/A
0 1.25 2.5 km
Wima-Robert-Downer- ID 90
0 1.5 3 km

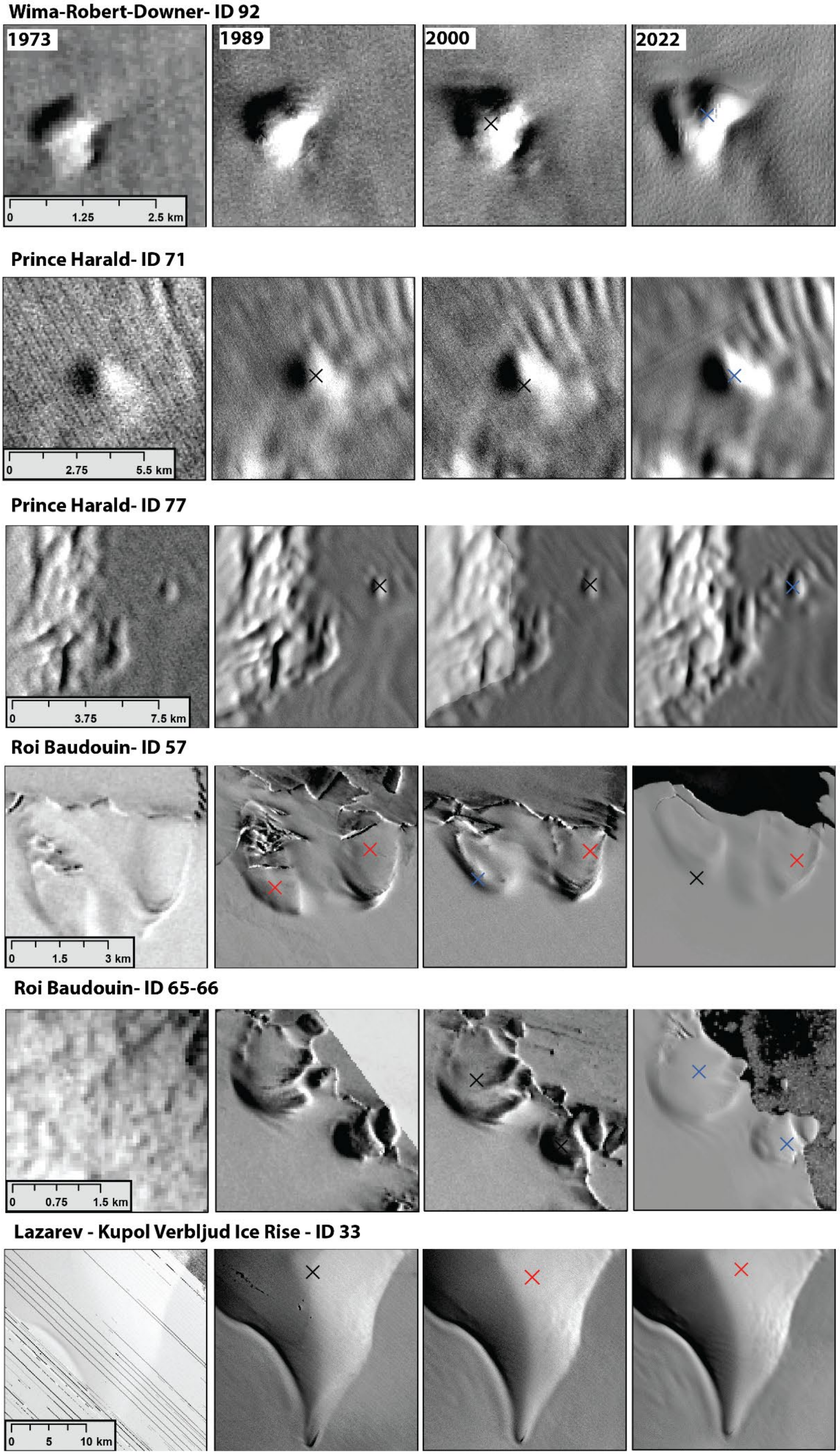

Wima-Robert-Downer- ID 92
1973
1989
2000
2022
0
1.25
2.5 km
Prince Harald- ID 71
0
2.75
5.5 km
Prince Harald- ID 77
0
3.75
7.5 km
Roi Baudouin- ID 57
0
1.5
3 km
Roi Baudouin- ID 65-66
0
0.75
1.5 km
Lazarev - Kupol Verbljud Ice Rise - ID 33
0
5
10 km

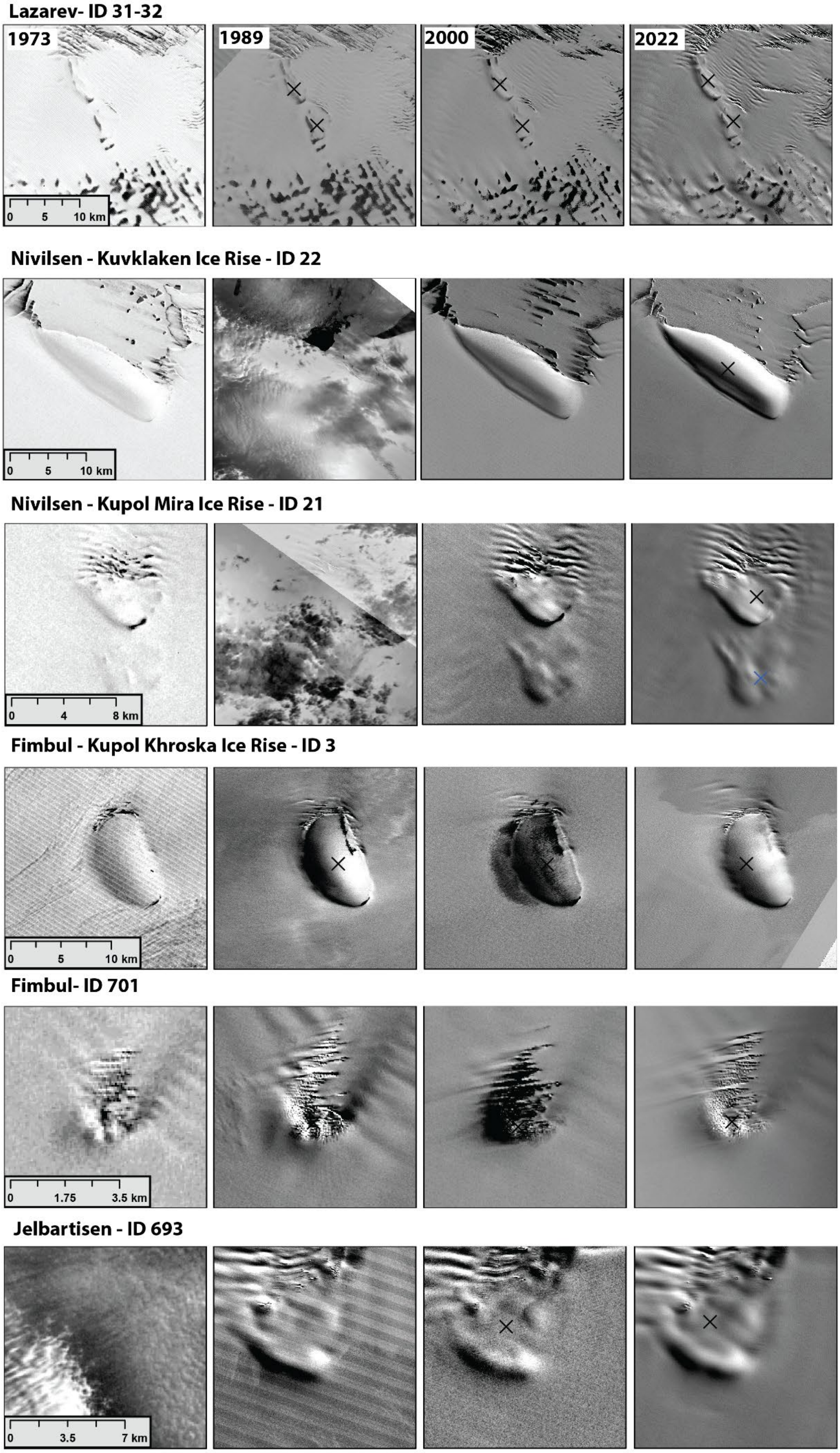
Lazarev- ID 31-32
1973
1989
2000
2022
0 5 10 km
Nivilsen - Kuvklaken Ice Rise - ID 22
0 5 10 km
Nivilsen - Kupol Mira Ice Rise - ID 21
0 4 8 km
Fimbul - Kupol Khroska Ice Rise - ID 3
0 5 10 km
Fimbul- ID 701
0 1.75 3.5 km
Jelbartisen - ID 693
0 3.5 7 km

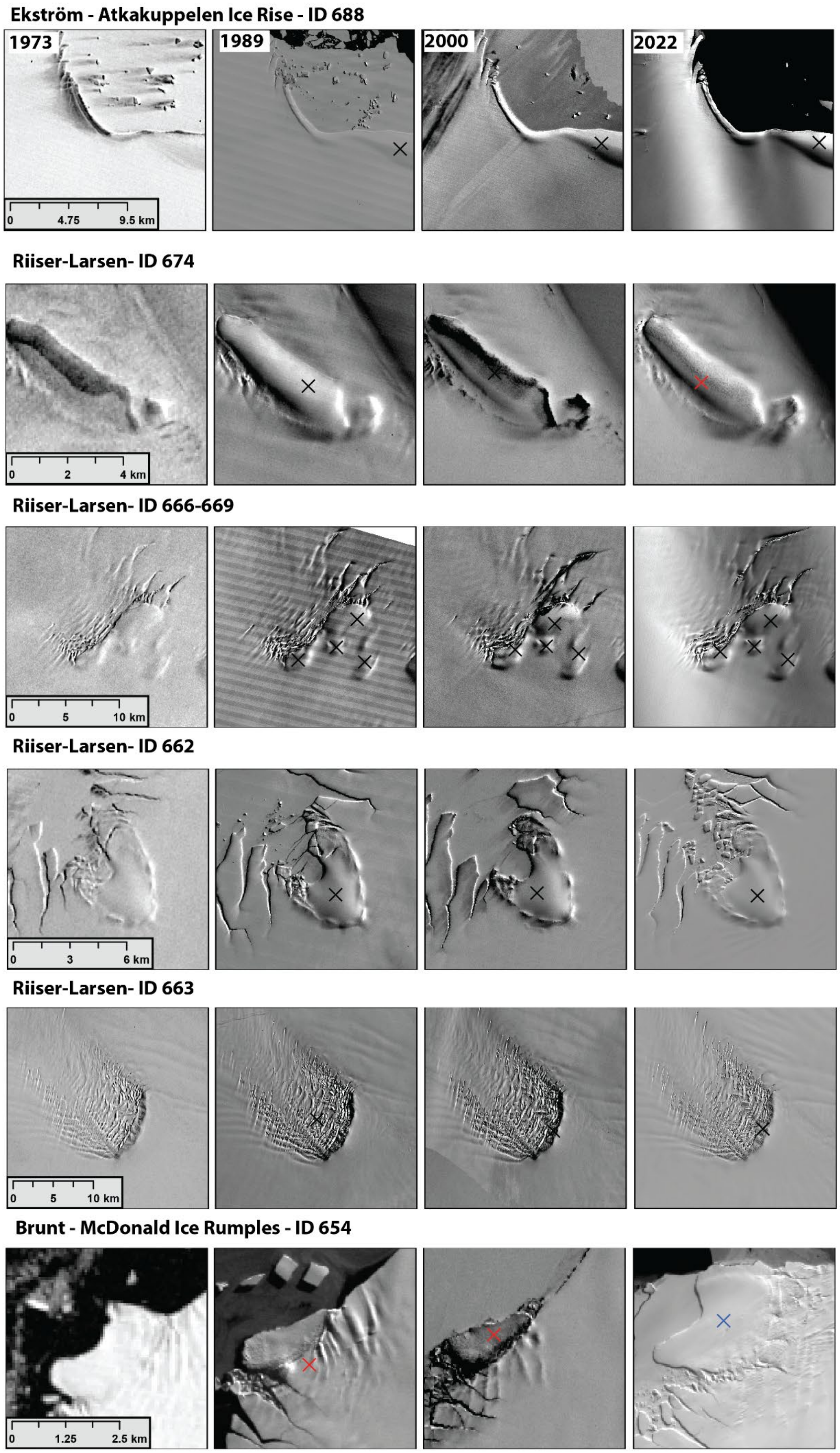
Ekström - Atkakuppelen Ice Rise - ID 688
1973
1989
2000
2022
0 4.75 9.5 km
Riiser-Larsen- ID 674
0 2 4 km
Riiser-Larsen- ID 666-669
0 5 10 km
Riiser-Larsen- ID 662
0 3 6 km
Riiser-Larsen- ID 663
0 5 10 km
Brunt - McDonald Ice Rumples - ID 654
0 1.25 2.5 km

Filchner–Ronne- ID 644-655

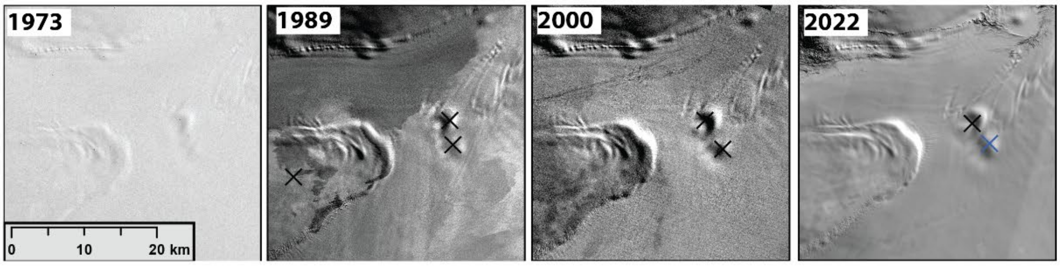

Filchner–Ronne- ID 641

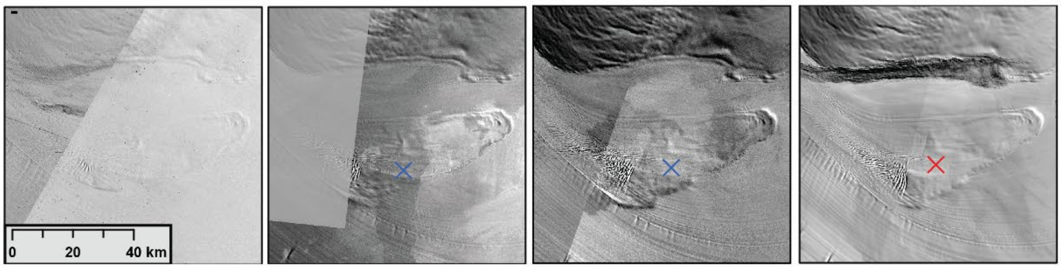

Filchner–Ronne- ID 642

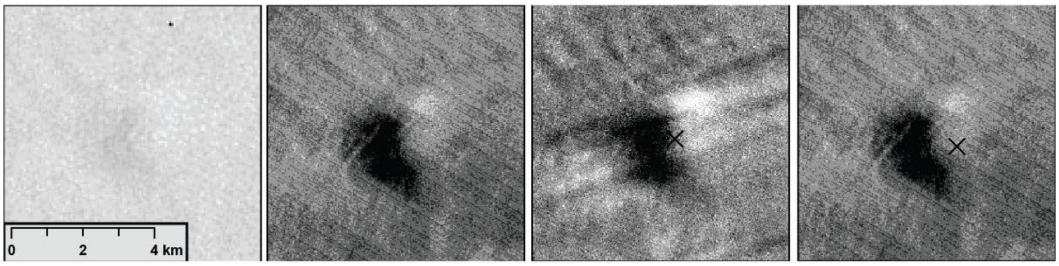

Filchner–Ronne - Doake Ice Rumples - ID 576 & 585

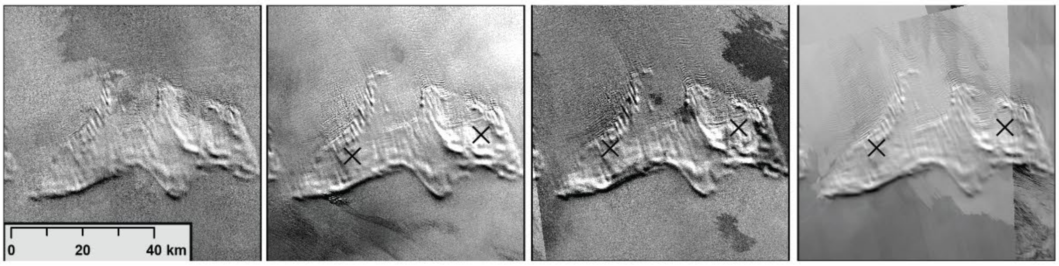

Filchner–Ronne- ID 471 & 474

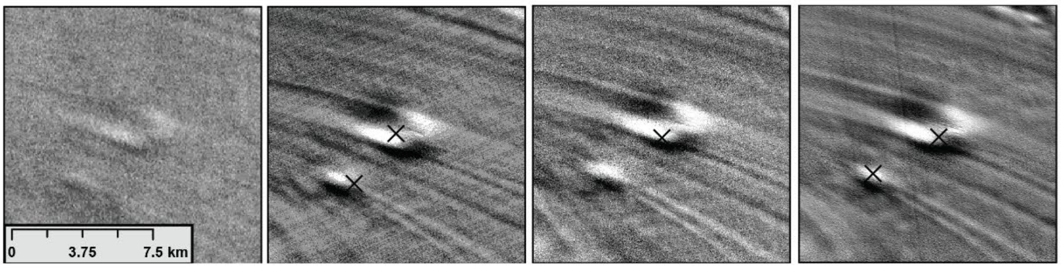

Filchner–Ronne - Kershaw Ice Rumples - ID 470

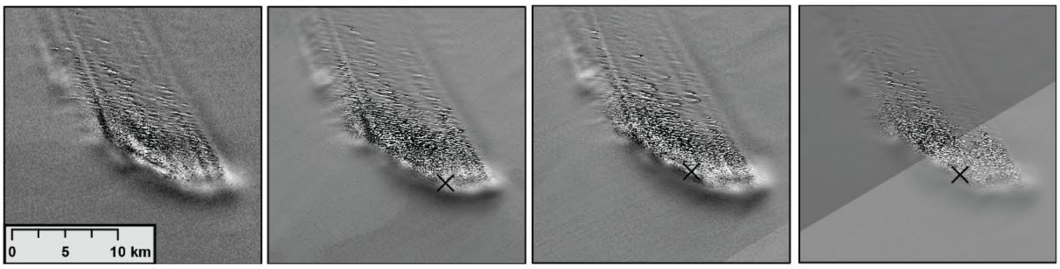

Landsat ID's used in 1973 mosaic	
LM01_L1GS_175119_19731105	LM01_L1GS_127107_19731115
LM01_L1GS_181118_19731116	LM01_L1GS_129107_19730115
LM01_L1GS_181119_19731116	LM01_L1GS_129108_19730115
LM01_L1GS_189119_19731211	LM01_L1GS_131107_19730204
LM01_L1GS_190117_19731211	LM01_L1GS_131108_19730204
LM01_L1GS_190118_19731211	LM01_L1GS_131109_19730204
LM01_L1GS_193119_19731110	LM01_L1GS_131110_19730204
LM01_L1GS_194117_19731110	LM01_L1GS_131111_19730204
LM01_L1GS_194118_19731110	LM01_L1GS_131112_19730204
LM01_L1GS_195116_19730127	LM01_L1GS_131113_19730204
LM01_L1GS_199119_19731110	LM01_L1GS_135111_19730316
LM01_L1GS_201116_19731117	LM01_L1GS_136111_19730227
LM01_L1GS_204116_19740113	LM01_L1GS_136112_19730227
LM01_L1GS_206116_19740202	LM01_L1GS_136113_19730227
LM01_L1GS_206118_19731210	LM01_L1GS_135112_19730316
LM01_L1GS_208118_19740204	LM01_L1GS_137109_19740223
LM01_L1GS_208119_19740204	LM01_L1GS_137110_19740223
LM01_L1GS_209117_19730211	LM01_L1GS_135109_19731211
LM01_L1GS_211115_19740207	LM01_L1GS_137111_19740223
LM01_L1GS_213115_19740209	LM01_L1GS_135110_19730316
LM01_L1GS_213118_19731217	LM01_L1GS_099107_19731104
LM01_L1GS_213119_19731217	LM01_L1GS_100107_19721215
LM01_L1GS_215116_19730217	LM01_L1GS_101107_19740117
LM01_L1GS_215117_19730217	LM01_L1GS_102107_19721219
LM01_L1GS_217116_19740213	LM01_L1GS_102108_19721219
LM01_L1GS_219114_19730220	LM01_L1GS_107107_19731026
LM01_L1GS_219115_19730220	LM01_L1GS_107108_19731026
LM01_L1GS_219116_19730220	LM01_L1GS_109106_19730131
LM01_L1GS_224115_19731210	LM01_L1GS_109107_19730131
LM01_L1GS_225114_19731210	LM01_L1GS_109108_19730131
LM01_L1GS_076109_19730220	LM01_L1GS_111107_19731117
LM01_L1GS_078109_19731101	LM01_L1GS_111108_19731117
LM01_L1GS_081109_19731104	LM01_L1GS_114107_19731015
LM01_L1GS_084108_19740118	LM01_L1GS_116106_19731104
LM01_L1GS_086108_19740207	LM01_L1GS_116107_19731104
LM01_L1GS_085107_19731214	LM01_L1GS_118106_19721129
LM01_L1GS_085108_19731214	LM01_L1GS_118107_19721129
LM01_L1GS_088107_19730304	LM01_L1GS_119107_19740223
LM01_L1GS_089107_19730128	LM01_L1GS_121107_19721202
LM01_L1GS_090107_19730129	LM01_L1GS_122106_19721202
LM01_L1GS_092107_19730218	LM01_L1GS_123106_19740227
LM01_L1GS_094107_19731030	LM01_L1GS_123107_19740227
LM01_L1GS_095107_19731013	LM01_L1GS_137108_19731231
LM01_L1GS_096107_19731101	LM01_L1GS_140108_19721221
LM01_L1GS_098106_19730101	LM01_L1GS_144107_19721207

LM01_L1GS_098107_19731103	LM01_L1GS_144108_19721207
LM01_L1GS_098107_19730101	LM01_L1GS_146108_19730219
LM01_L1GS_124107_19730215	LM01_L1GS_147107_19730202
LM01_L1GS_126107_19731114	LM01_L1GS_147108_19730202
LM01_L1GS_126108_19731114	LM01_L1GS_150107_19740218
LM01_L1GS_150108_19740218	LM01_L1GS_247112_19730107
LM01_L1GS_153108_19740116	LM02_L1GS_236112_19750218
LM01_L1GS_154107_19740117	LM03_L1GS_233110_19781229
LM01_L1GS_154108_19740117	LM01_L1GS_161110_19731113
LM01_L1GS_156108_19740101	LM01_L1GS_161111_19731113
LM01_L1GS_156109_19731214	LM01_L1GS_161112_19731113
LM01_L1GS_156110_19731214	LM01_L1GS_162110_19731114
LM01_L1GS_158109_19740121	LM01_L1GS_163109_19731115
LM01_L1GS_161109_19731113	LM01_L1GS_163110_19731115
LM01_L1GS_162109_19731114	LM01_L1GS_164110_19731116
LM01_L1GS_177110_19731111	LM01_L1GS_164111_19731116
LM01_L1GS_179110_19731113	LM01_L1GS_164112_19731116
LM01_L1GS_179111_19731113	LM01_L1GS_165109_19740128
LM01_L1GS_182110_19731204	LM01_L1GS_165110_19731117
LM01_L1GS_182111_19731204	LM01_L1GS_166109_19731118
LM01_L1GS_183111_19730310	LM01_L1GS_167110_19731119
LM01_L1GS_186110_19731102	LM01_L1GS_167111_19731119
LM01_L1GS_190113_19740222	LM01_L1GS_168110_19731120
LM01_L1GS_190114_19740222	LM01_L1GS_168111_19731120
LM01_L1GS_191111_19740118	LM01_L1GS_170110_19731210
LM01_L1GS_191112_19740118	LM02_L1GS_173109_19751019
LM01_L1GS_191113_19740118	LM02_L1GS_173110_19751124
LM01_L1GS_192114_19721218	LM02_L1GS_175110_19751126
LM01_L1GS_192115_19721218	LM02_L1GS_177109_19760208
LM01_L1GS_195112_19730127	LM01_L1GS_012119_19731107
LM01_L1GS_195114_19730127	LM01_L1GS_017119_19731107
LM01_L1GS_197113_19730110	LM01_L1GS_018114_19731219
LM01_L1GS_197113_19730216	LM01_L1GS_018118_19731219
LM02_L1GS_182111_19751028	LM01_L1GS_021114_19730114
LM02_L1GS_184110_19751030	LM01_L1GS_021115_19730114
LM02_L1GS_189110_19750225	LM01_L1GS_022115_19721228
LM02_L1GS_190110_19751123	LM01_L1GS_022116_19721228
LM01_L1GS_002111_19730113	LM01_L1GS_027113_19721214
LM01_L1GS_228112_19740206	LM01_L1GS_027116_19721214
LM01_L1GS_231112_19730109	LM01_L1GS_030116_19721129
LM01_L1GS_232110_19730109	LM01_L1GS_030117_19731126
LM01_L1GS_232111_19730109	LM01_L1GS_032117_19731127
LM01_L1GS_232113_19730109	LM01_L1GS_033115_19721203
LM01_L1GS_233109_19740106	LM02_L1GS_018119_19751130
LM01_L1GS_233111_19730129	LM02_L1GS_023114_19751205
LM01_L1GS_233112_19730129	LM02_L1GS_025115_19760217

LM01_L1GS_233113_19730129	LM02_L1GS_025116_19760217
LM01_L1GS_235113_19730112	LM01_L1GS_046118_19740116
LM01_L1GS_236109_19740109	LM02_L1GS_051116_19751109
LM01_L1GS_237110_19721121	LM01_L1GS_046119_19740116
LM01_L1GS_237111_19721121	LM01_L1GS_050118_19731127
LM01_L1GS_237113_19730115	LM01_L1GS_051116_19721221
LM01_L1GS_240112_19740131	LM01_L1GS_051116_19740103
LM01_L1GS_243112_19730121	LM01_L1GS_051117_19740103
LM01_L1GS_247111_19730107	LM01_L1GS_052116_19740104
LM01_L1GS_052117_19740104	LM02_L1GS_249113_19750213
LM01_L1GS_053116_19740105	LM01_L1GS_006113_19721230
LM01_L1GS_053117_19740105	LM01_L1GS_010113_19731123
LM01_L1GS_054116_19721224	LM01_L1GS_010114_19731123
LM01_L1GS_055117_19730130	LM01_L1GS_014114_19731127
LM01_L1GS_055117_19740107	LM01_L1GS_018113_19730111
LM01_L1GS_056116_19730113	LM01_L1GS_019113_19730111
LM01_L1GS_056116_19740108	LM01_L1GS_019114_19730111
LM01_L1GS_056117_19740126	LM01_L1GS_020113_19721120
LM01_L1GS_057116_19730113	LM01_L1GS_020114_19721120
LM01_L1GS_058116_19731223	LM01_L1GS_022113_19730114
LM01_L1GS_064114_19730102	LM01_L1GS_246114_19730124
LM01_L1GS_064114_19740116	LM01_L1GS_248114_19730213
LM01_L1GS_065111_19721128	LM01_L1GS_004114_19730116
LM01_L1GS_065112_19721128	
LM01_L1GS_065113_19721128	
LM01_L1GS_065114_19721128	
LM01_L1GS_066114_19740117	
LM01_L1GS_067110_19721201	
LM01_L1GS_067111_19721201	
LM01_L1GS_067112_19721201	
LM01_L1GS_068110_19730125	
LM01_L1GS_068111_19721202	
LM01_L1GS_070111_19730108	
LM01_L1GS_071110_19721205	
LM01_L1GS_072110_19731026	
LM01_L1GS_073110_19740124	
LM02_L1GS_042117_19751206	
LM02_L1GS_050116_19751021	
LM01_L1GS_001112_19721207	
LM01_L1GS_001112_19730130	
LM01_L1GS_001113_19730130	
LM01_L1GS_001114_19730130	
LM01_L1GS_002113_19730113	
LM01_L1GS_003112_19721227	
LM01_L1GS_003113_19721227	
LM01_L1GS_003114_19730113	

Landsat ID’s used in 1973 mosaic	
LT04_L1GS_038116_19880221	LT05_L1GS_082108_19890102
LT05_L1GS_156110_19861203	LT04_L1GS_088106_19890205
LT04_L1GS_155108_19891110	LT04_L1GS_088107_19890205
LT04_L1GS_155109_19891110	LT04_L1GS_087107_19890318
LT04_L1GS_157109_19890124	LT04_L1GS_084107_19900127
LT04_L1GS_157110_19891108	LT04_L1GS_084108_19900127
LT04_L1GS_157111_19891108	LT04_L1GS_092106_19891218
LT04_L1GS_155110_19891212	LT04_L1GS_092107_19891218
LT04_L1GS_159109_19900125	LT04_L1GS_090106_19900121
LT04_L1GS_163109_19891204	LT04_L1GS_090107_19900121
LT04_L1GS_163110_19891204	LT05_L1GS_080108_19890104
LT04_L1GS_163111_19891204	LT05_L1GS_094107_19910213
LT04_L1GS_161109_19890325	LT04_L1GS_045116_19891225
LT04_L1GS_161110_19890325	LT04_L1GS_045117_19891225
LT04_L1GS_165109_19880215	LT04_L1GS_045118_19891225
LT04_L1GS_153109_19891128	LT04_L1GS_045119_19891225
LT04_L1GS_159110_19900125	LT04_L1GS_050116_19890227
LT04_L1GS_167109_19880128	LT04_L1GS_055115_19881212
LT04_L1GS_167110_19880128	LT04_L1GS_035119_19900120
LT04_L1GS_217103_19890128	LT04_L1GS_035120_19900120
LT04_L1GS_221108_19891124	LT04_L1GS_050115_19900129
LT04_L1GS_215103_19890319	LT04_L1GS_050117_19900129
LT05_L1GS_217106_19860301	LT04_L1GS_050118_19900129
LT04_L1GS_219105_19891126	LT04_L1GS_050119_19900129
LT05_L1GS_217107_19860301	LT04_L1GS_055116_19890129
LT05_L1GS_217108_19860301	LT04_L1GS_055117_19890129
LT05_L1GS_217105_19860301	LT04_L1GS_060113_19881215
LT04_L1GS_217104_19890128	LT04_L1GS_060114_19881215
LT04_L1GS_219104_19901215	LT04_L1GS_060115_19881215
LT04_L1GS_215104_19880229	LT04_L1GS_062112_19891114
LT04_L1GS_215105_19880229	LT04_L1GS_062113_19900117
LT04_L1GS_219106_19891126	LT04_L1GS_062114_19900117
LT04_L1GS_210111_19890127	LT04_L1GS_062115_19900117
LT04_L1GS_210112_19890127	LT04_L1GS_064110_19891128
LT04_L1GS_214108_19880206	LT04_L1GS_065111_19891119
LT04_L1GS_215107_19880112	LT04_L1GS_066109_19891228
LT04_L1GS_215109_19880128	LT04_L1GS_066110_19891228
LT04_L1GS_219108_19891212	LT04_L1GS_068110_19900111
LT04_L1GS_219109_19891212	LT04_L1GS_068111_19900111
LT05_L1GS_219107_19890219	LT04_L1GS_070109_19890207
LT04_L1GS_215108_19880128	LT04_L1GS_070110_19890207
LT04_L1GS_217109_19900115	LT05_L1GS_062111_19910128
LT04_L1GS_213110_19890321	LT04_L1GS_105107_19891112
LT05_L1GS_220107_19860218	LT04_L1GS_096106_19891231
LT05_L1GS_074109_19910217	LT04_L1GS_096107_19891231

LT04_L1GS_072109_19890221	LT04_L1GS_096108_19891231
LT04_L1GS_076108_19891116	LT04_L1GS_098107_19900130
LT04_L1GS_076109_19891116	LT04_L1GS_100107_19891227
LT04_L1GS_078108_19890303	LT04_L1GS_102107_19891107
LT05_L1GS_082107_19890102	LT04_L1GS_102108_19891107
LT04_L1GS_104107_19891121	LT04_L1GS_024113_19881219
LT04_L1GS_105106_19891112	LT04_L1GS_232113_19900124
LT04_L1GS_114105_19890228	LT04_L1GS_012113_19880114
LT04_L1GS_114106_19890228	LT04_L1GS_021113_19880214
LT04_L1GS_114107_19891111	LT04_L1GS_004113_19890209
LT04_L1GS_110106_19891115	LT04_L1GS_006113_19900109
LT04_L1GS_110107_19891115	LT04_L1GS_004114_19890209
LT04_L1GS_108107_19890218	LT04_L1GS_008112_19891222
LT04_L1GS_112106_19891113	LT04_L1GS_024112_19881219
LT04_L1GS_112107_19891113	LT04_L1GS_139107_19880124
LT05_L1GS_116106_19910208	LT04_L1GS_130108_19890316
LT04_L1GS_169109_19891214	LT04_L1GS_133108_19900220
LT04_L1GS_173109_19891108	LT04_L1GS_135107_19891114
LT04_L1GS_173110_19900228	LT04_L1GS_135108_19891114
LT04_L1GS_177109_19891120	LT04_L1GS_137107_19891112
LT05_L1GS_176109_19871015	LT04_L1GS_137108_19900131
LT04_L1GS_171109_19900214	LT04_L1GS_141106_19891124
LT05_L1GS_183113_19860130	LT04_L1GS_141107_19900212
LT04_L1GS_185114_19890317	LT04_L1GS_143106_19890327
LT04_L1GS_170110_19891221	LT04_L1GS_143107_19890327
LT04_L1GS_170111_19891221	LT04_L1GS_143108_19890327
LT04_L1GS_169110_19891214	LT04_L1GS_145107_19900312
LT04_L1GS_171110_19900113	LT04_L1GS_145108_19900312
LT04_L1GS_173111_19900228	LT04_L1GS_147108_19880217
LT04_L1GS_179110_19900121	LT04_L1GS_148108_19890125
LT04_L1GS_179111_19900206	LT04_L1GS_149109_19880215
LT04_L1GS_177110_19871030	LT04_L1GS_151108_19890215
LT04_L1GS_181111_19900119	LT04_L1GS_151109_19890215
LT04_L1GS_181112_19900119	LT04_L1GS_151110_19890215
LT04_L1GS_185113_19890317	LT04_L1GS_149108_19880114
LT04_L1GS_181114_19900220	LT04_L1GS_153108_19880110
LT04_L1GS_183111_19900117	LT04_L1GS_183116_19900101
LT04_L1GS_183113_19900117	LT04_L1GS_185116_19890301
LT04_L1GS_183115_19900101	LT04_L1GS_189117_19900127
LT04_L1GS_181113_19900119	LT04_L1GS_190115_19890216
LT05_L1GS_176110_19871015	LT04_L1GS_190117_19890131
LT04_L1GS_183112_19891114	LT04_L1GS_190118_19890131
LT04_L1GS_021112_19890115	LT04_L1GS_195115_19891204
LT04_L1GS_018112_19890211	LT04_L1GS_195116_19891204
LT04_L1GS_018113_19890211	LT04_L1GS_199114_19890130
LT04_L1GS_015113_19900124	LT04_L1GS_199115_19890130

LT04_L1GS_001112_19891221	LT04_L1GS_190116_19890131
LT04_L1GS_001113_19891221	LT04_L1GS_207113_19880308
LT04_L1GS_004112_19891226	LT04_L1GS_207114_19880308
LT04_L1GS_008113_19900123	LT04_L1GS_207115_19880308
LT04_L1GS_004113_19880122	LT04_L1GS_207116_19880308
LT04_L1GS_004114_19880122	LT04_L1GS_210116_19890127
LT04_L1GS_010112_19891220	LT04_L1GS_210117_19890127
LT04_L1GS_010113_19891220	LT04_L1GS_213113_19890201
LT04_L1GS_015112_19900124	LT04_L1GS_213114_19890201
LT04_L1GS_213115_19890116	LT04_L1GS_125109_19891226
LT04_L1GS_213116_19890201	LT04_L1GS_221109_19900127
LT04_L1GS_213117_19890321	LT04_L1GS_219112_19891212
LT04_L1GS_215115_19890130	LT04_L1GS_215112_19881229
LT04_L1GS_215116_19890130	LT04_L1GS_215111_19881229
LT05_L1GS_169118_19860301	LT04_L1GS_221111_19891124
LT05_L1GS_169119_19860301	LT04_L1GS_221110_19900127
LT05_L1GS_169120_19860213	LT04_L1GS_217111_19890128
LT05_L1GS_169121_19860213	LT04_L1GS_219110_19891126
LT05_L1GS_180117_19860226	LT04_L1GS_219111_19891126
LT05_L1GS_180118_19860226	LT04_L1GS_218110_19890220
LT05_L1GS_180119_19860226	LT05_L1GS_219109_19910124
LT05_L1GS_183119_19860215	LT04_L1GS_223111_19891224
LT05_L1GS_183120_19860215	LT04_L1GS_004111_19891226
LT05_L1GS_183121_19860215	LT04_L1GS_226112_19880210
LT05_L1GS_199116_19860215	LT04_L1GS_229112_19890201
LT05_L1GS_199117_19860215	LT05_L1GS_232111_19910220
LT05_L1GS_199118_19860215	LT04_L1GS_001111_19891221
LT05_L1GS_202115_19860220	LT04_L1GS_221112_19890124
LT05_L1GS_202116_19860220	LT04_L1GS_221113_19890124
LT05_L1GS_204116_19860306	LT04_L1GS_223112_19891224
LT05_L1GS_204117_19860306	LT04_L1GS_229111_19890201
LT05_L1GS_204118_19860306	LT04_L1GS_229112_19890217
LT04_L1GS_187117_19890227	LT05_L1GS_232112_19910220
LT04_L1GS_204114_19891219	LT04_L1GS_233123_19891128
LT04_L1GS_190120_19890131	LT04_L1GS_021119_19891115
LT04_L1GS_190119_19890131	LT04_L1GS_007118_19881212
LT04_L1GS_185117_19890301	LT04_L1GS_233122_19891128
LT04_L1GS_128108_19891129	LT04_L1GS_030115_19900218
LT04_L1GS_128109_19891129	LT05_L1GS_016119_19870115
LT04_L1GS_128110_19891129	LT04_L1GS_014117_19900218
LT04_L1GS_128111_19891129	LT05_L1GS_025117_19870114
LT04_L1GS_128112_19891129	LT04_L1GS_021118_19900203
LT04_L1GS_116107_19890314	LT04_L1GS_025117_19891229
LT04_L1GS_118107_19890312	LT04_L1GS_019118_19890117
LT04_L1GS_120106_19900124	LT04_L1GS_014118_19900218
LT04_L1GS_120107_19900124	LT04_L1GS_007119_19881212

LT04_L1GS_122107_19891205	LT04_L1GS_024114_19881219
LT04_L1GS_122108_19891205	LT04_L1GS_024115_19881219
LT04_L1GS_124107_19900120	LT04_L1GS_024116_19890309
LT04_L1GS_124108_19900120	LT04_L1GS_025119_19891229
LT04_L1GS_124109_19900120	LT04_L1GS_025118_19891229
LT04_L1GS_120108_19900124	LT04_L1GS_030114_19900218
LT04_L1GS_125110_19891108	LT04_L1GS_030117_19900202
LT04_L1GS_125111_19891108	LT04_L1GS_035116_19900205
LT04_L1GS_125112_19900212	LT04_L1GS_035117_19900205
LT04_L1GS_125113_19900212	LT04_L1GS_030116_19900202
LT04_L1GS_128113_19891231	
LT04_L1GS_129110_19890309	
LT04_L1GS_131109_19891118	
