# Supplementary figures and images for: Progressive unanchoring of Antarctic ice shelves since 1973
Source: Nature. 2024 Feb 21;626(8000):785–91. doi: 10.1038/s41586-024-07049-0 (PMC10881387; doi:10.1038/s41586-024-07049-0)

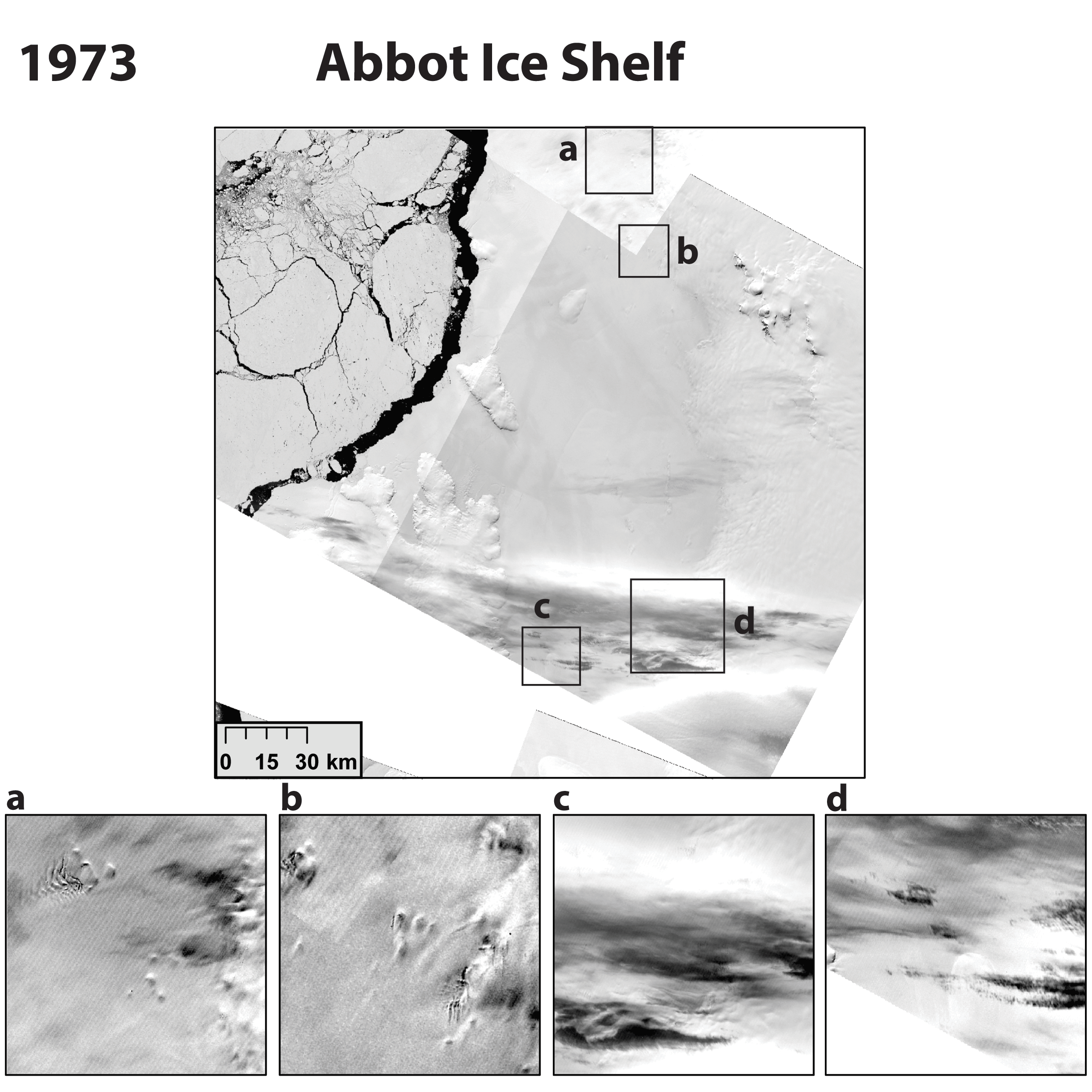

Supplement: Supplementary file 3 — Animated images of pinning-point change [file 41586_2024_7049_MOESM3_ESM.zip › Abbot_IceShelf.gif]

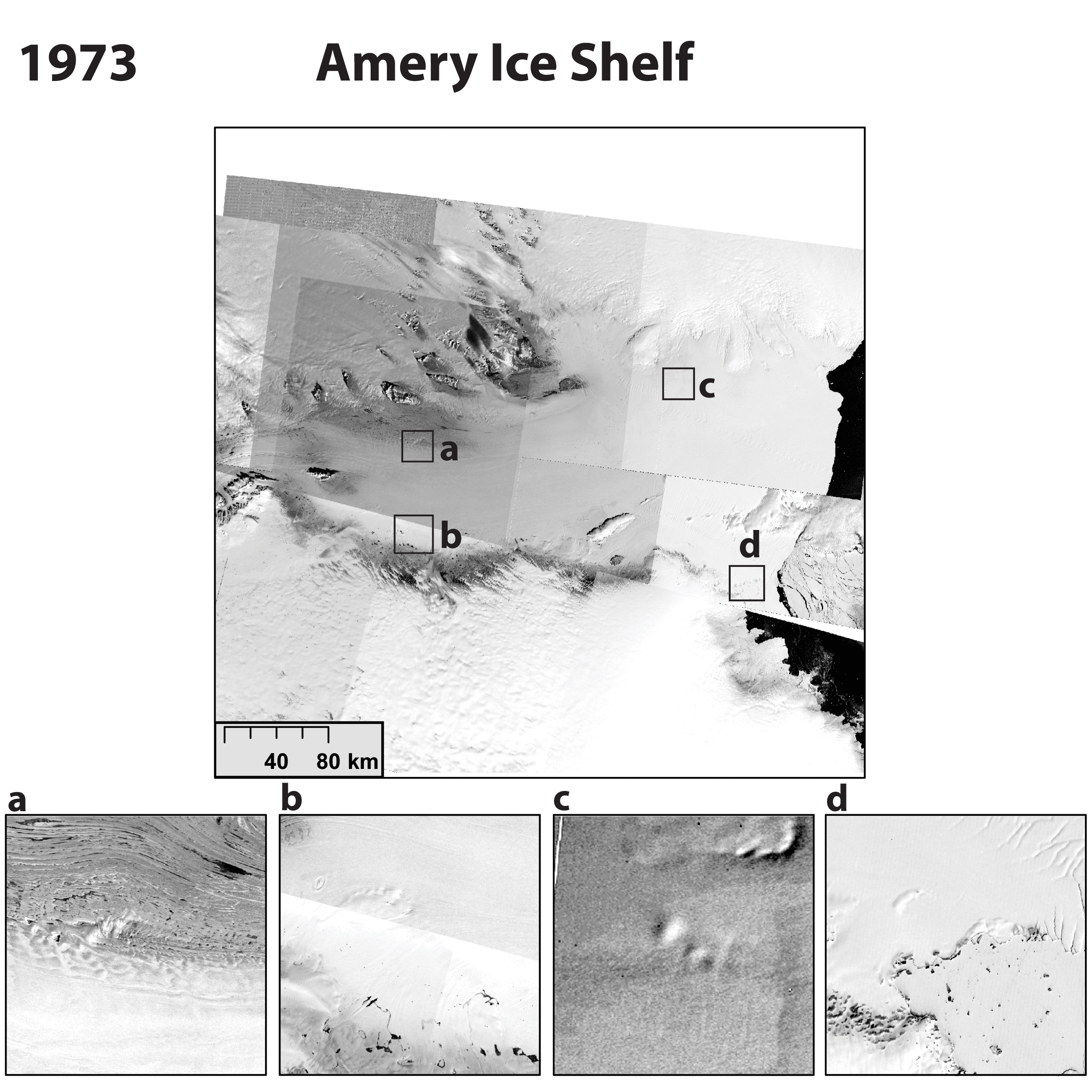

Supplement: Supplementary file 3 — Animated images of pinning-point change [file 41586_2024_7049_MOESM3_ESM.zip › Amery_IceShelf.gif]

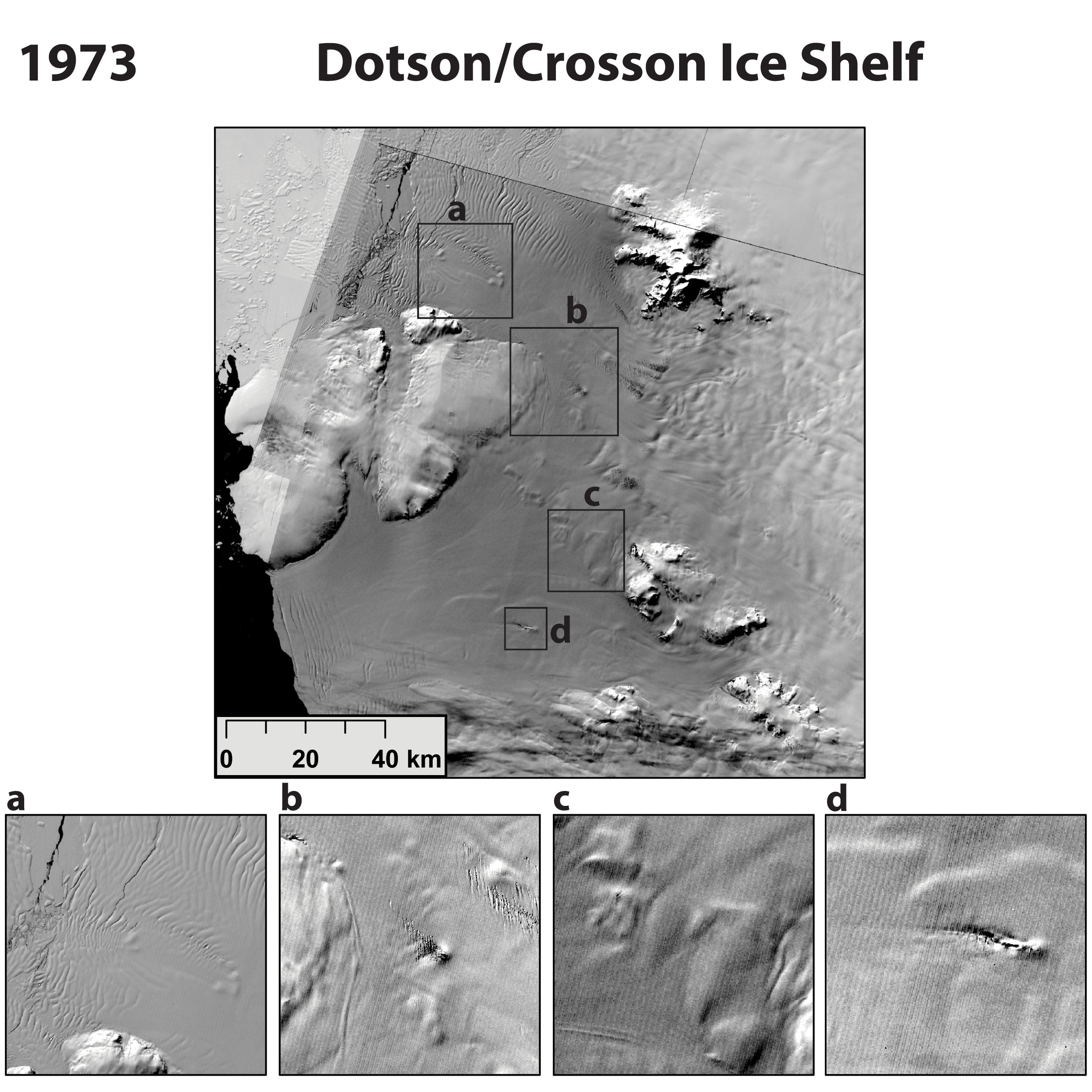

Supplement: Supplementary file 3 — Animated images of pinning-point change [file 41586_2024_7049_MOESM3_ESM.zip › DotsonCrosson_IceShelf.gif]

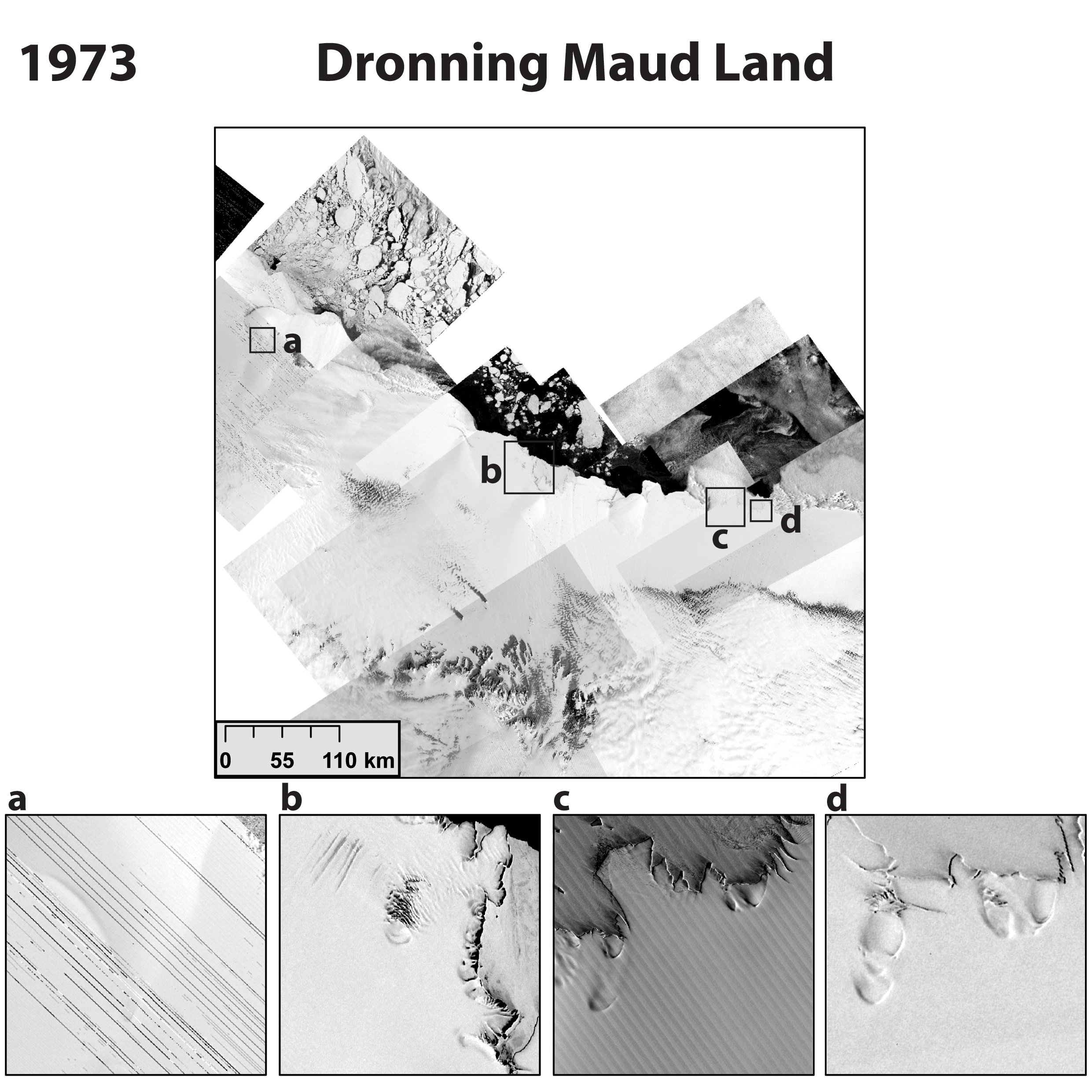

Supplement: Supplementary file 3 — Animated images of pinning-point change [file 41586_2024_7049_MOESM3_ESM.zip › DronningMaudLand.gif]

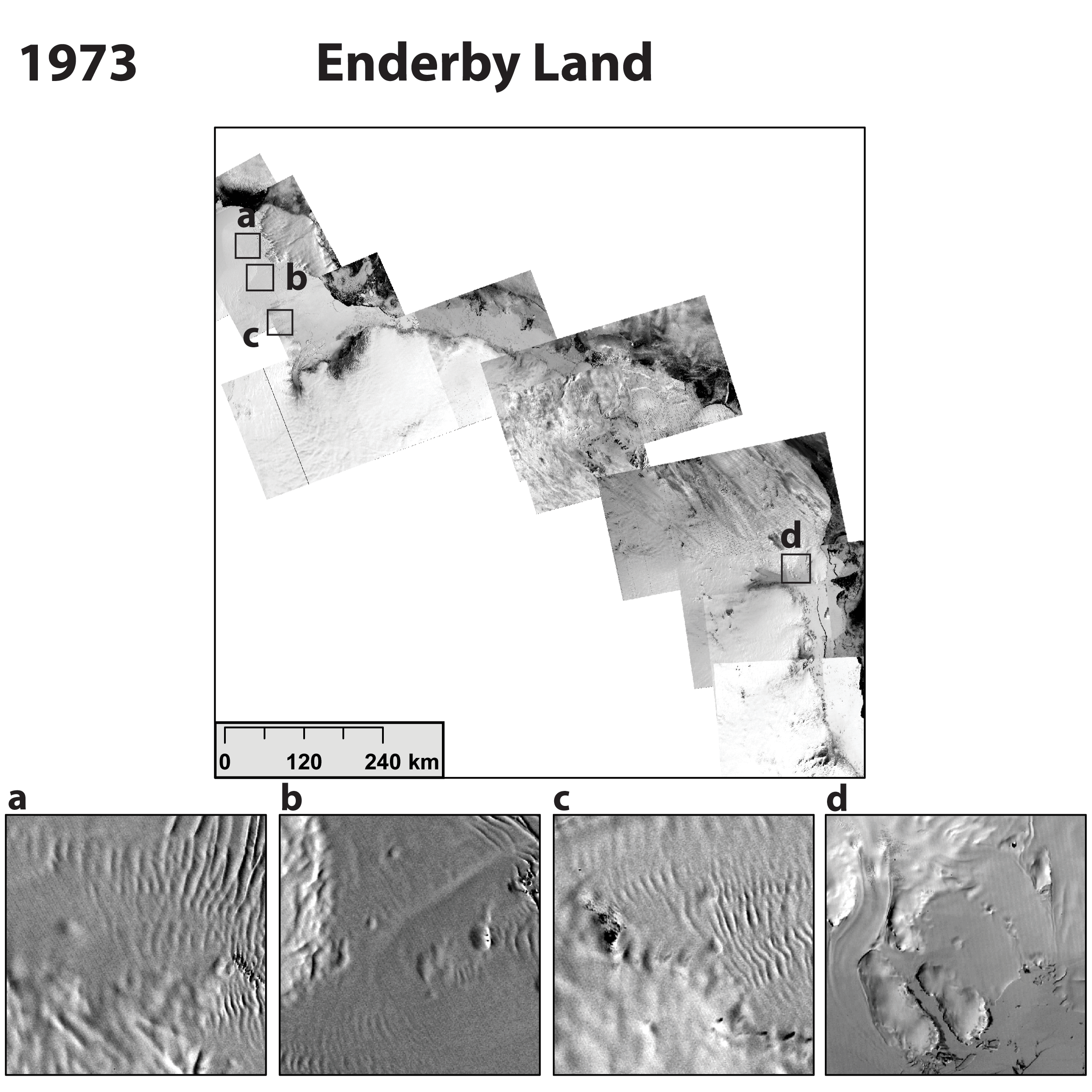

Supplement: Supplementary file 3 — Animated images of pinning-point change [file 41586_2024_7049_MOESM3_ESM.zip › EnderbyLand.gif]

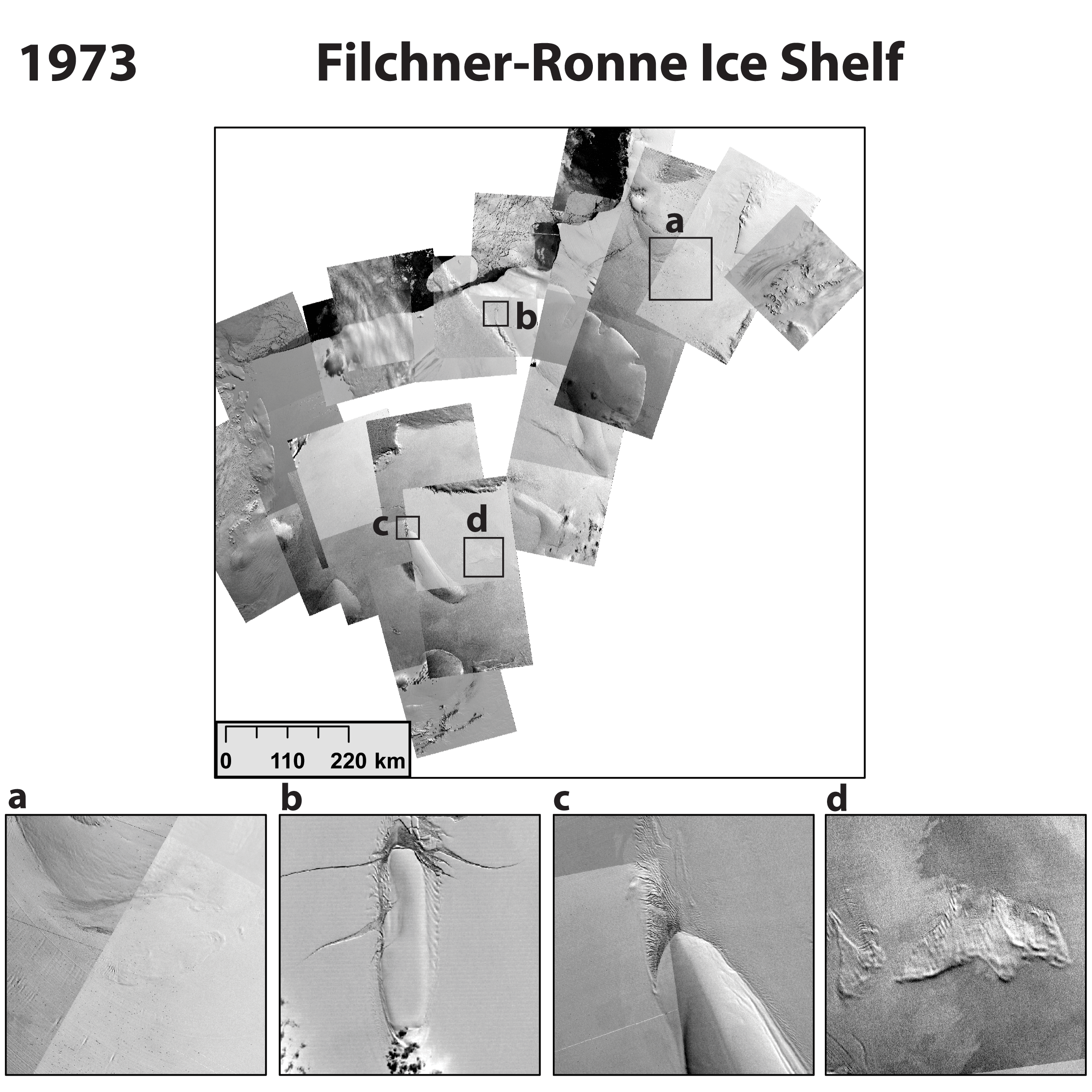

Supplement: Supplementary file 3 — Animated images of pinning-point change [file 41586_2024_7049_MOESM3_ESM.zip › FR_IceShelf.gif]

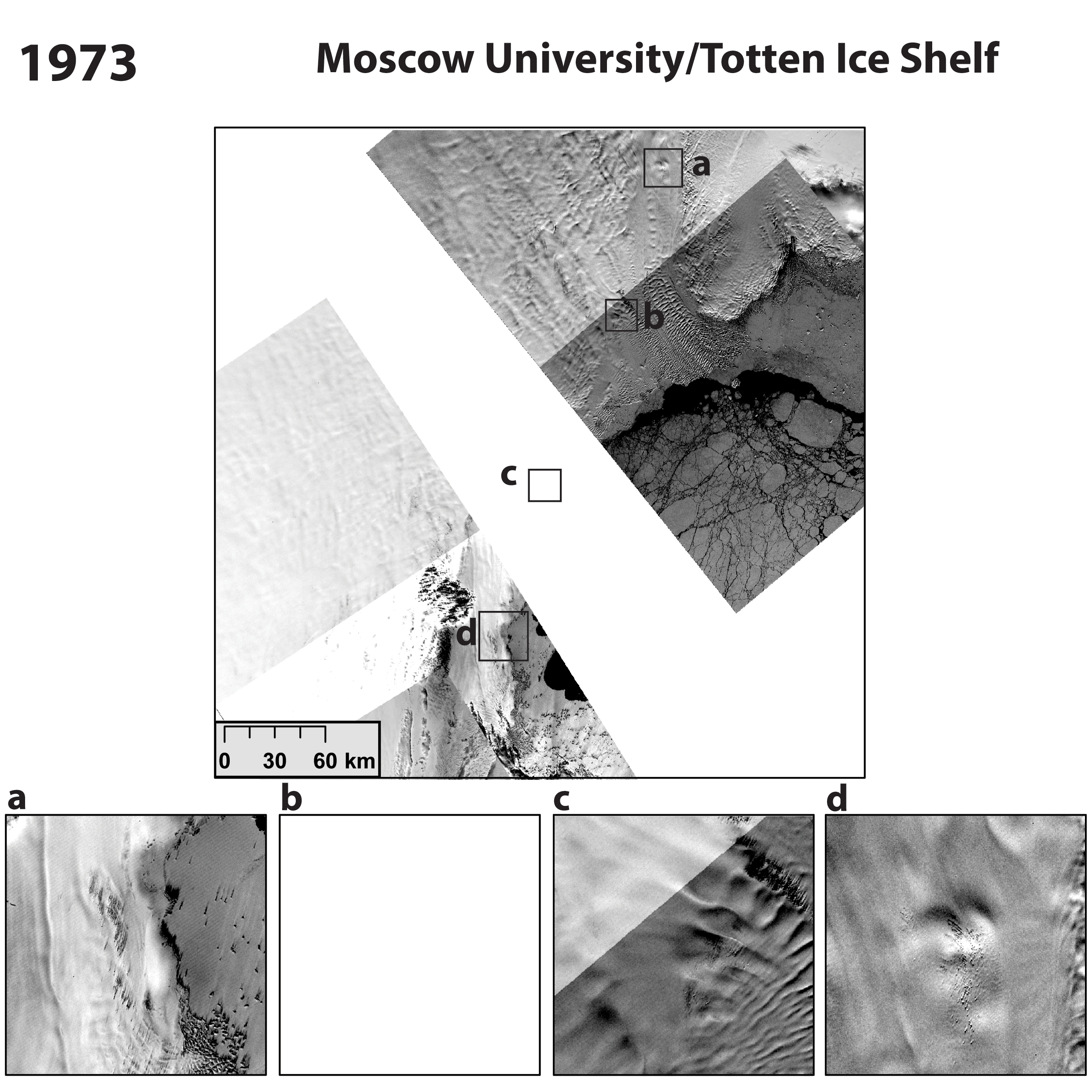

Supplement: Supplementary file 3 — Animated images of pinning-point change [file 41586_2024_7049_MOESM3_ESM.zip › MoscowTotten_IceShelf.gif]

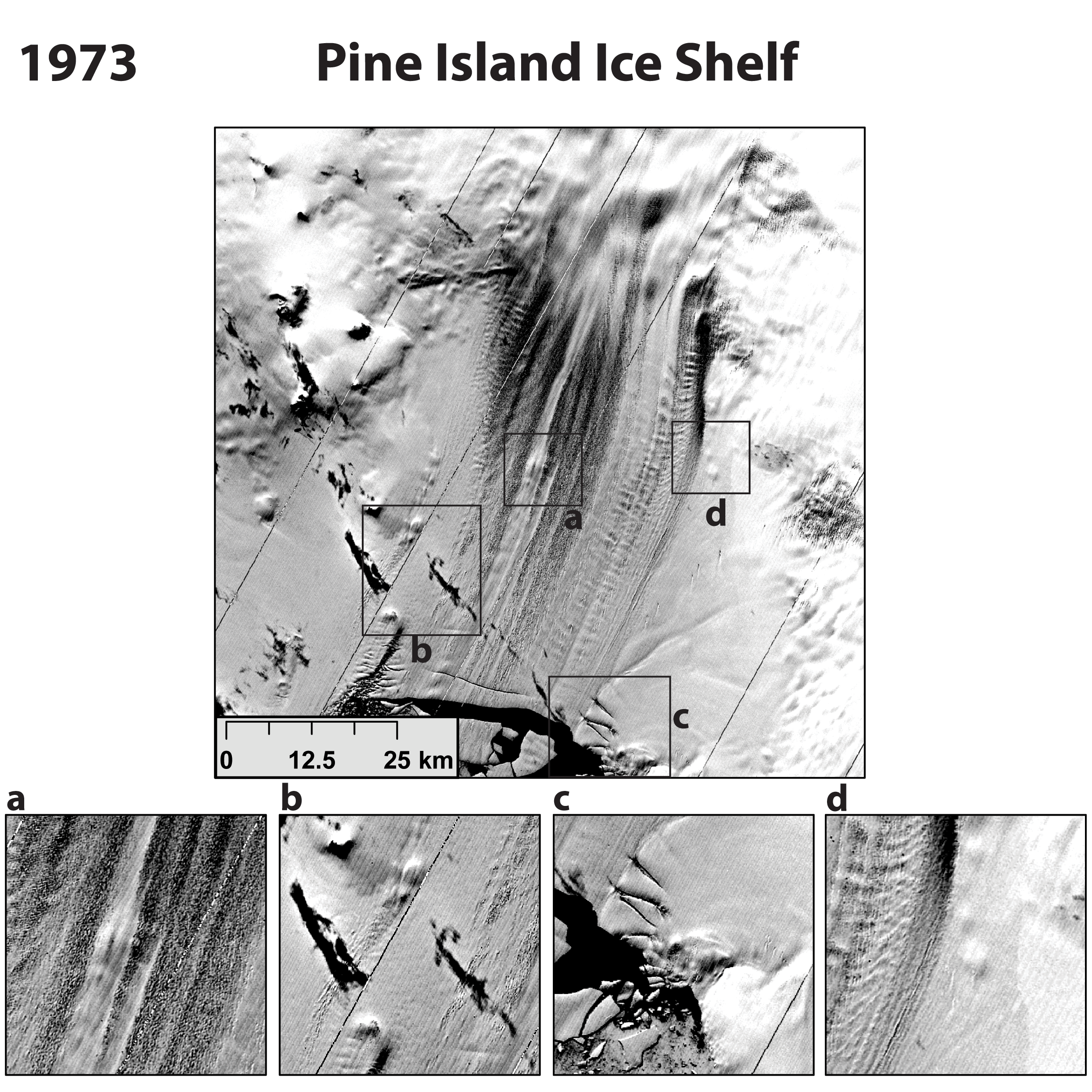

Supplement: Supplementary file 3 — Animated images of pinning-point change [file 41586_2024_7049_MOESM3_ESM.zip › Pine_Island_IceShelf.gif]

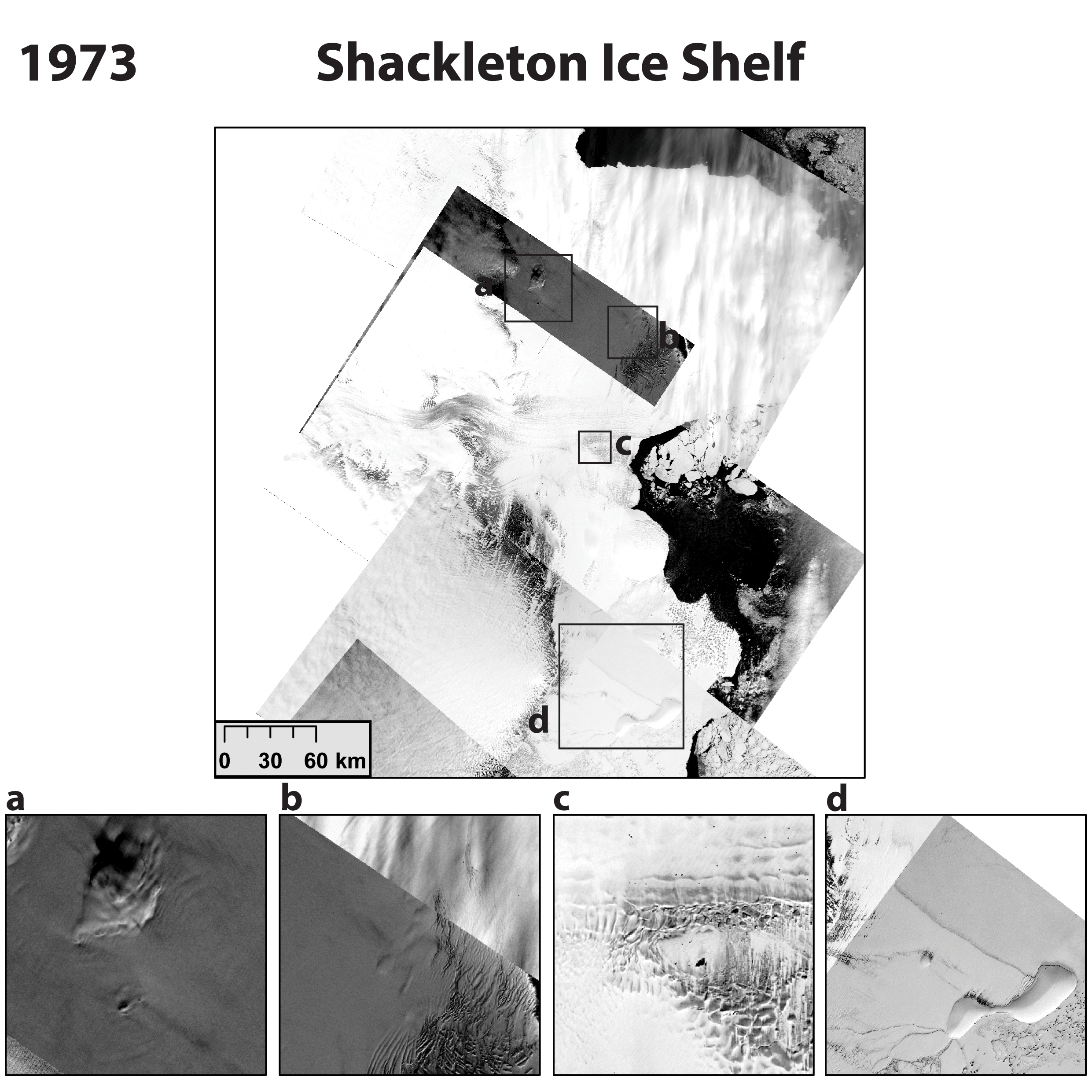

Supplement: Supplementary file 3 — Animated images of pinning-point change [file 41586_2024_7049_MOESM3_ESM.zip › Shackleton_IceShelf.gif]

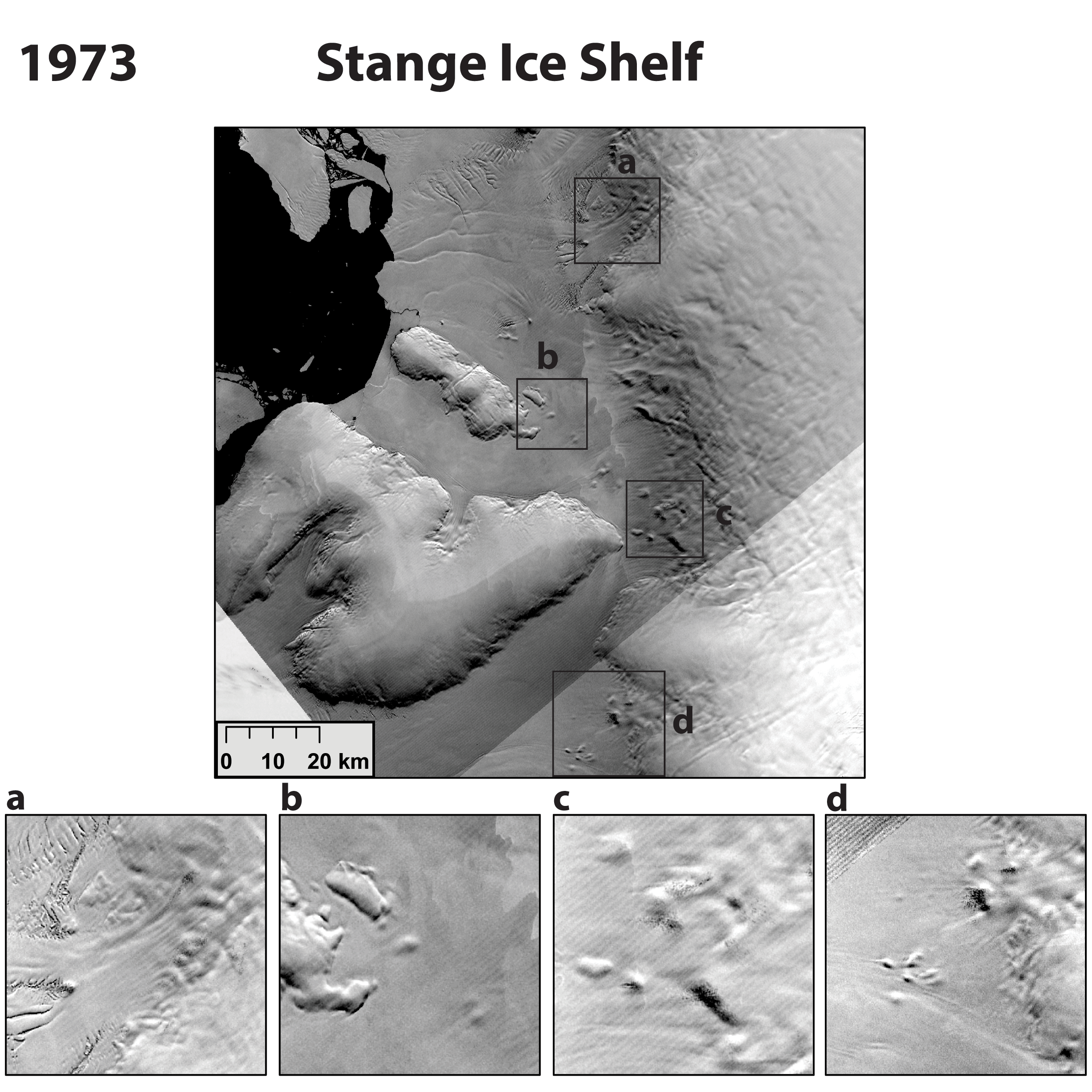

Supplement: Supplementary file 3 — Animated images of pinning-point change [file 41586_2024_7049_MOESM3_ESM.zip › Stange_IceShelf.gif]

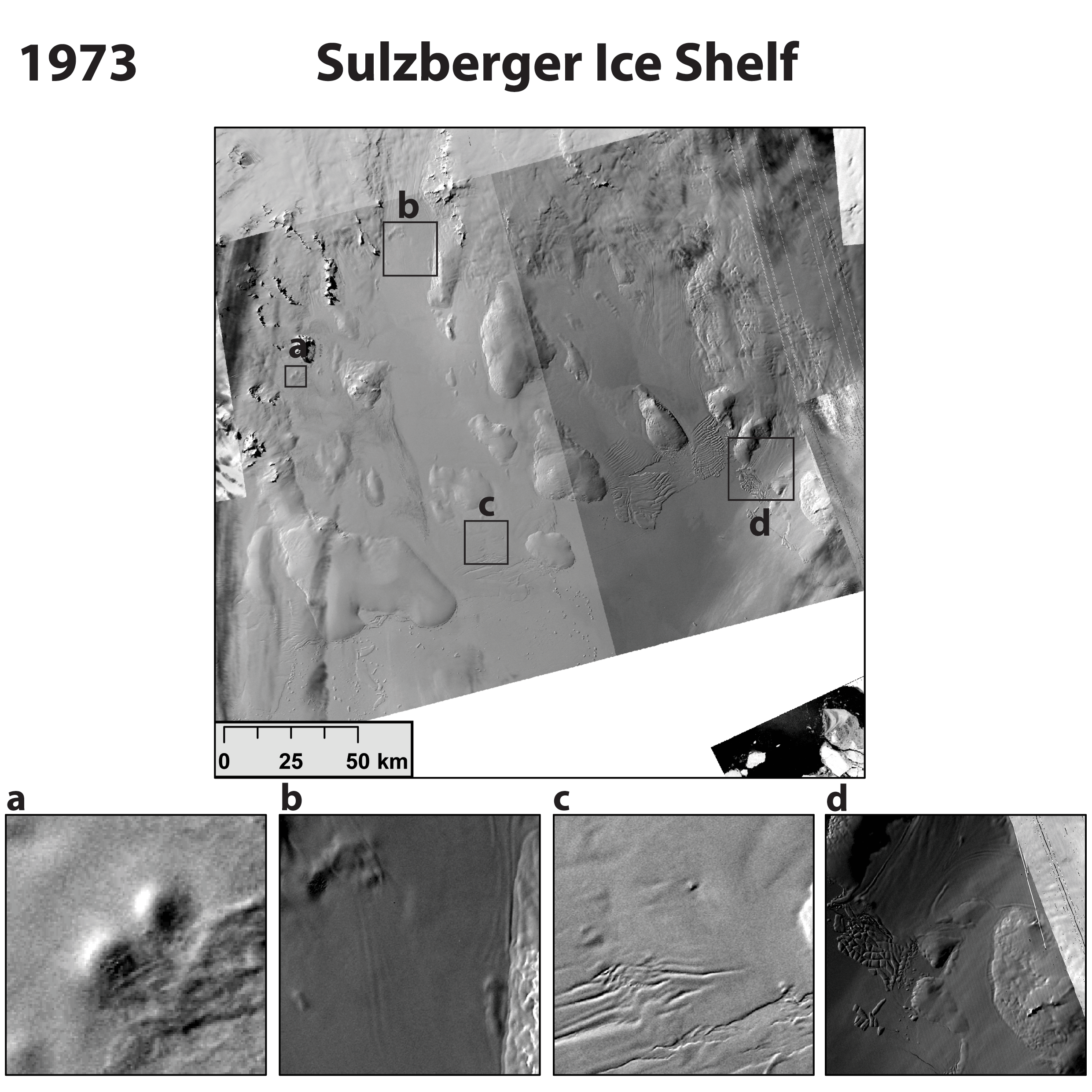

Supplement: Supplementary file 3 — Animated images of pinning-point change [file 41586_2024_7049_MOESM3_ESM.zip › Sulzberger_IceShelf.gif]

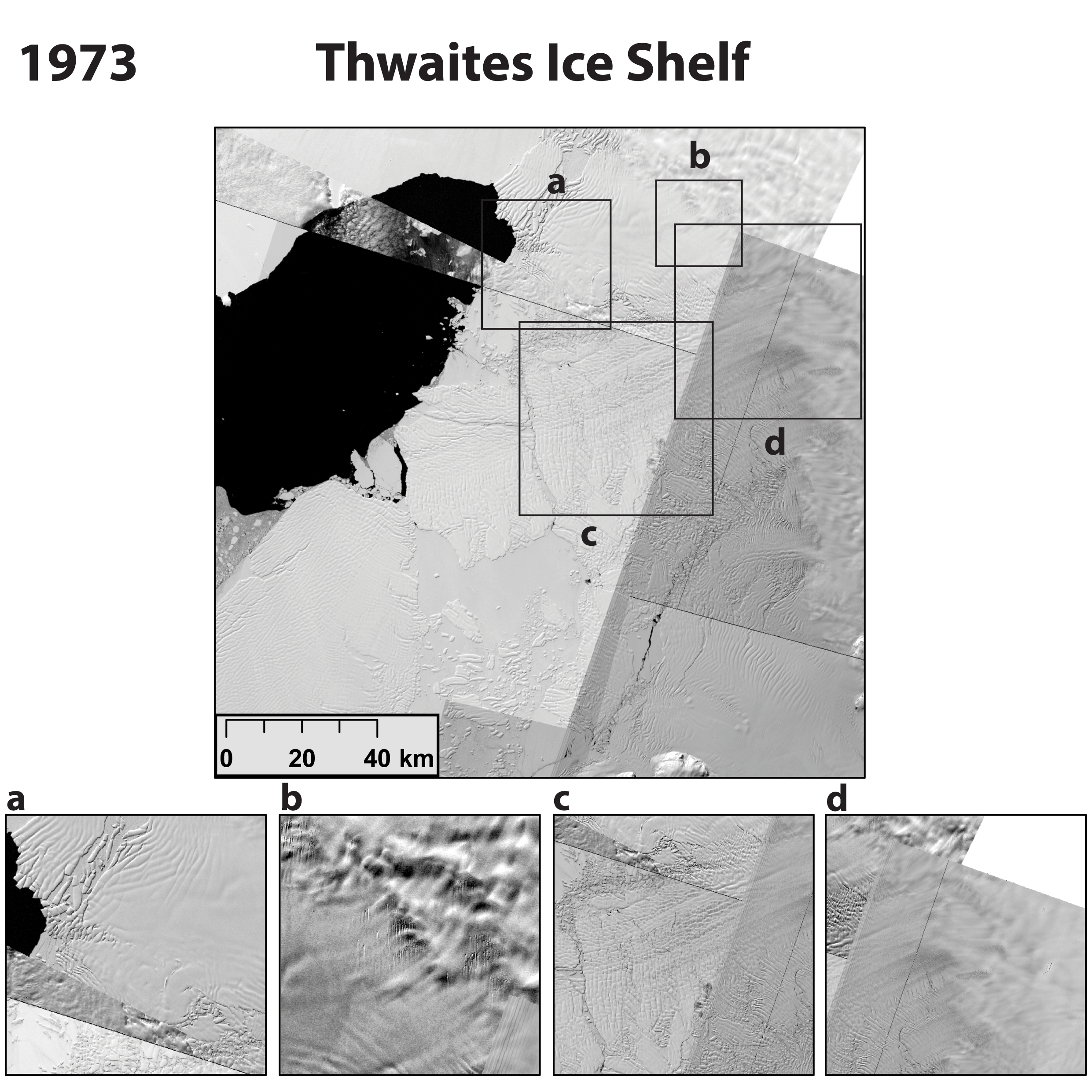

Supplement: Supplementary file 3 — Animated images of pinning-point change [file 41586_2024_7049_MOESM3_ESM.zip › Thwaites_IceShelf.gif]

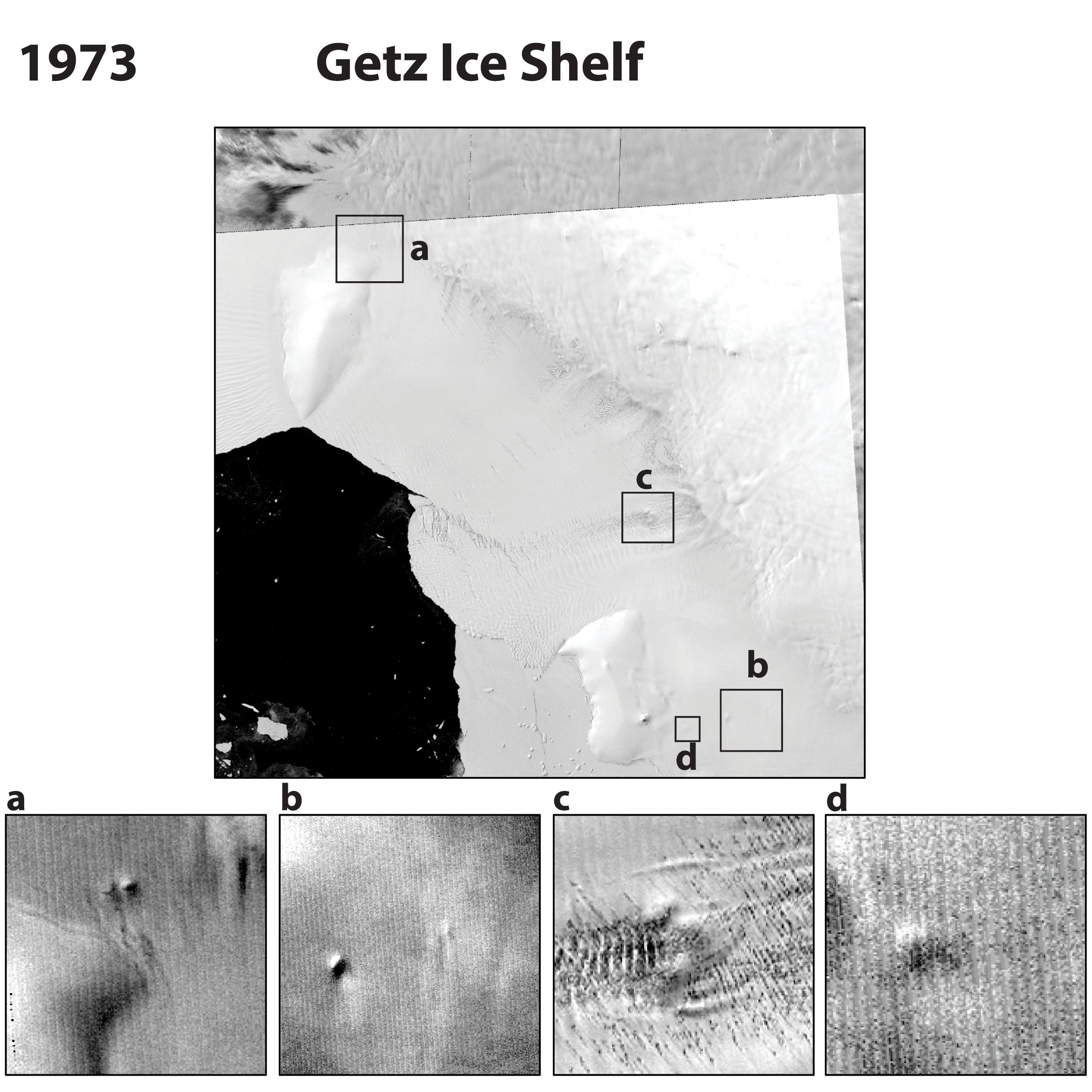

Supplement: Supplementary file 3 — Animated images of pinning-point change [file 41586_2024_7049_MOESM3_ESM.zip › WesternGetz_IceShelf.gif]

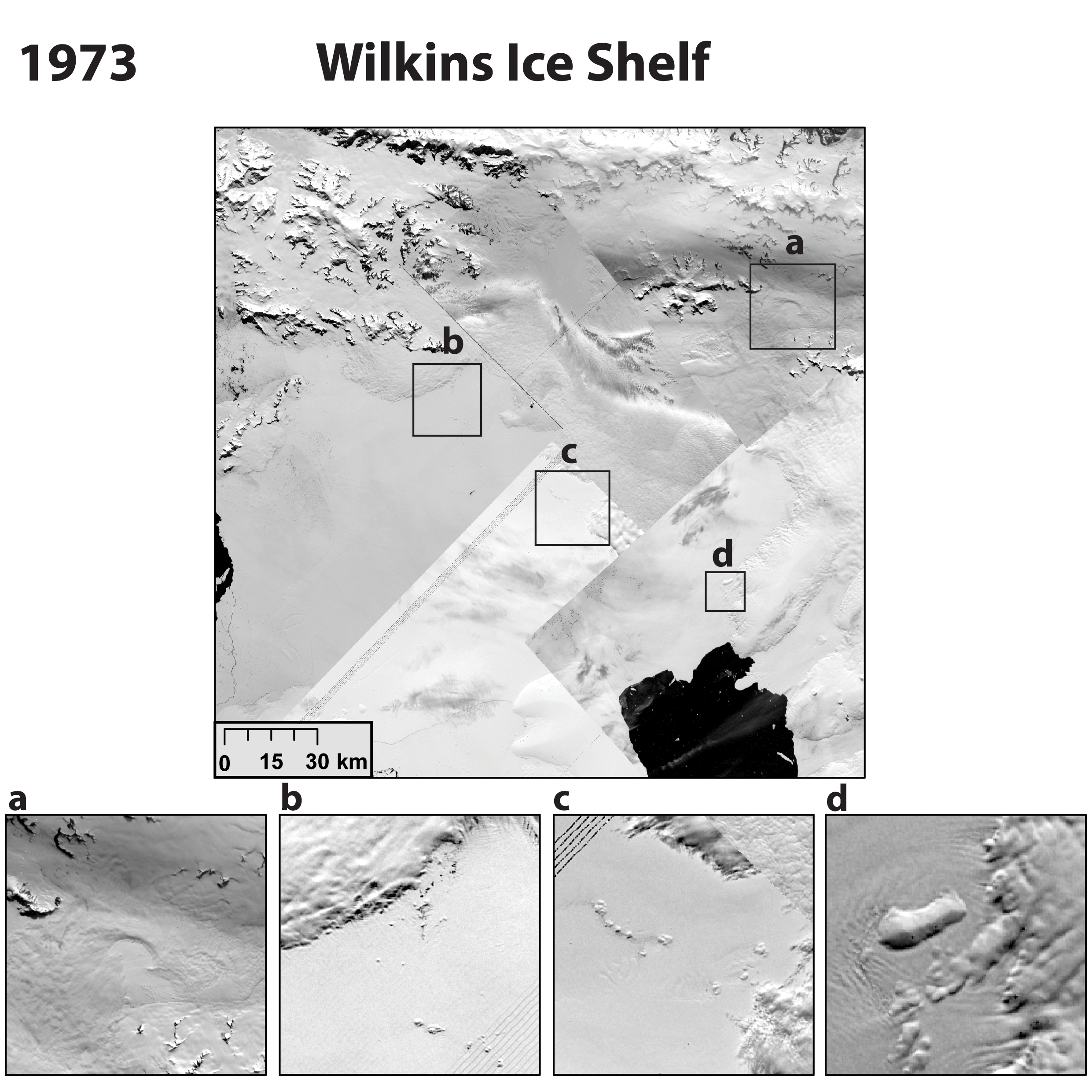

Supplement: Supplementary file 3 — Animated images of pinning-point change [file 41586_2024_7049_MOESM3_ESM.zip › Wilkins_IceShelf.gif]
